# Combination strategies to overcome drug resistance in FLT^+^ acute myeloid leukaemia

**DOI:** 10.1186/s12935-023-03000-x

**Published:** 2023-08-11

**Authors:** Jingmei Yang, Ran Friedman

**Affiliations:** Department of Chemistry and Biomedical Science, Linnaeus University, Kalmar Campus, 391 82 Kalmar, Sweden

**Keywords:** Combination treatment, FLT3-ITD, Targeted therapy, Alpelisib, Duvelisib, Idelalisib, Copanlisib, Drug synergism, Gilteritinib, Acute myeloid leukemia

## Abstract

**Background:**

Acute myeloid leukaemia (AML) remains difficult to treat despite the development of novel formulations and targeted therapies. Activating mutations in the FLT3 gene are common among patients and make the tumour susceptible to FLT3 inhibitors, but resistance to such inhibitors develops quickly.

**Methods:**

We examined combination therapies aimed at FLT3^+^-AML, and studied the development of resistance using a newly developed protocol. Combinations of FLT3, CDK4/6 and PI3K inhibitors were tested for synergism.

**Results:**

We show that AML cells express CDK4 and that the CDK4/6 inhibitors palbociclib and abemaciclib inhibit cellular growth. PI3K inhibitors were also effective in inhibiting the growth of AML cell lines that express FLT3-ITD. Whereas resistance to quizartinib develops quickly, the combinations overcome such resistance.

**Conclusions:**

This study suggests that a multi-targeted intervention involving a CDK4/6 inhibitor with a FLT3 inhibitor or a pan-PI3K inhibitor might be a valuable therapeutic strategy for AML to overcome drug resistance. Moreover, many patients cannot tolerate high doses of the drugs that were studied (quizartinib, palbociclib and PI3K inhibitors) for longer periods, and it is therefore of high significance that the drugs act synergistically and lower doses can be used.

**Supplementary Information:**

The online version contains supplementary material available at 10.1186/s12935-023-03000-x.

## Introduction

Acute myeloid leukaemia (AML) is a genetically diverse haematopoietic malignancy [1]. In spite of newer drugs and better understanding of disease mechanisms, progress leading to better survival has been slow, especially for females and older patients (age >75) [2]. Of the many genetic variations that have prognostic significance for AML patients, FLT3 mutations are very common (30% of such patients, according to current estimations [3]). These mutations make the FLT3 tyrosine kinase (that is normally important in haematopoietic stem and progenitor cells) overactive. In particular, the so called internal tandem duplications [4] activate the protein by disturbing a regulatory interaction [4, 5]. Several agents exist that inhibit FLT3 including two FDA and EMA approved drugs (midostaurin and gilteritinib) and quizartinib, a highly specific FLT3 inhibitor [6] approved in USA and Japan. Although useful, these inhibitors, and many others that are being developed are subject to development of drug resistance [7]. Resistance occurs via multiple mechanisms, of which mutations in the drug target is one of the most common [8–10].

Even in FLT3^+^-AML, other signalling proteins play an important role. Cyclin dependent kinases (CDKs) are critical to the regulation of cell cycle and gene expression, making them important drug targets [11]. CDK4 and the closely related CDK6 are activated by binding to D-type cyclins, which lead to the irreversible progression of the cell cycle from G1 to S phase. Many studies demonstrated the usefulness of CDK4/6 as a therapeutic target for cancer treatment. A CDK4/6 inhibitor, palbociclib, is currently approved for treatment of breast cancer, and showed significant cytotoxicity to FLT3-ITD AML cells [12]. Abemaciclib and ribociclib are other clinically approved CDK4/6 inhibitors [11] with similarity in their efficacy and side-effects. Palbociclib is robust to resistance mutations [13] and has been suggested as a useful AML  drug, especially in combination with additional agents [12, 14–16]. The efficacy of CDK4/6 inhibitors in AML is largely attributed to inhibiting CDK6 [12, 15, 17].

Many studies have demonstrated that targeting signalling downstream of FLT3 has a potential to overcome the drug resistance. Aberrant phosphatidylinositol 3-kinase (PI3K)/Akt/mammalian target of rapamycin (mTOR) pathway has been implicated in AML cells [18, 19] and promotes cell proliferation, growth, and survival. Of the various PI3K, the p110$\delta$ PI3K isoform is known to be overactive in AML [20]. Several PI3K inhibitors are approved for clinical use and others are under development, making them of interest even for AML [21].

Given that the high activity of quizartinib against AML is overcome by development of resistance, we previously examined whether switching between FLT3 inhibitors with different means of resistance (different resistance mutations) could be a useful approach to prolong resistance-free survival in AML. Unfortunately, although such approach seemed promising in CML [22], it did not work in AML [23]. Here, we examined whether combination therapy might be more useful. To this end, we studied whether the CDK4/6 inhibitors palbociclib and abemaciclib, and PI3K inhibitors are effective against AML cell lines expressing activating FLT3-ITD mutants (MOLM-13, MOLM-14 and MV4-11 cell lines; an additonal cell line expressing wild type FLT3 is also used in some experiments). Thereafter, we examined combinations of FLT3, CDK4/6 and PI3K inhibitors, with two aims: (1) to view if there is synergism between the drugs, i.e., if the two drugs are more effective together than expected from their individual contributions [24] and (2) to follow on the development of resistance, using our newly developed protocol to study resistance in cell lines [23].

The PI3K inhibitors that were studied include idelalisib, duvelisib, copanlisib and alpelisib. Idelalisib is an orally available, highly selective PI3K$\delta$ inhibitor that is primarily used for haematological malignancies [25–28]. Duvelisib is a potent PI3K$\delta$ and PI3K$\gamma$ inhibitor [29], and was studied here to examine if the dual PI3K$\gamma \delta$ inhibition is more effective. Copanlisib is a pan-PI3K inhibitor, with activity against the $\alpha$, $\beta$, $\gamma$ and $\delta$ isoforms [30], and was included to examine the activity of non-selective inhibitors. Finally, although the role of the PI3K$\alpha$ isoform in AML has not been established, we wished to examine if a clinically available PI3K$\alpha$ inhibitor, alpelisib [31] might also be useful in AML, especially since it is used for a more common form of cancer (metastatic breast cancer) than the other PI3K inhibitors.

## Materials and methods

### Reagents

Cell culture medium (RPMI 1640, Iscove’s Modified Dulbecco’s Medium—IMDM) was purchased from Fisher Scientific. PI3K and CDK6 inhibitors (idelalisinb, duvelisib, alpelisib, copanlisib and palbociclib) were also purchased from Fisher Scientific. Quizartinib was obtained from AdipoGen Life Sciences. Gilteritinib was purchased from Cayman Chemical Company. Abemaciclib was purchased from Selleck Chemicals. Each inhibitor was dissolved in dimethyl sulfoxide (DMSO). Pierce Protease Inhibitor Mini Tablets were purchased from Fisher Scientific. MTS (3-(4,5-dimethylthiazol-2-yl)-5-(3-carboxymethoxyphenyl)-2-(4-sulfophenyl)-2*H*-tetrazolium) was purchased from Promega.

### Cell cultures

MOLM-13, MOLM-14 and MV4-11 were a generous gift from Prof. Stefan Fröhling, National Center for Tumor Diseases, Germany. MV4-11 cells were cultured in IMDM supplemented with 10% (vol/vol) fetal bovine serum (FBS; Fisher Scientific, Sweden) and 1% (vol/vol) antibiotics (Penicillin–Streptomycin, Gibco, Fisher Scientific, Sweden). MOLM-13 and MOLM-14 were cultured in RPMI-1640 medium supplemented with 10% (vol/vol) FBS and 1% (vol/vol) antibiotics (Penicillin–Streptomycin). All cells were cultured in a humidified incubator at 37 °C with 5% CO_2_ and maintained at a density of 0.5 × 10^6^ to 1.5 × 10^6^ cells/mL by splitting the cultures every 2 to 3 days. FLT3 wild-type cell line Kasumi-1 cells were purchased from Cell Line Service (CLS), and were maintained in RPMI-1640 medium supplemented with 10% FBS, 1% (vol/vol) antibiotics and 2.5 mM l-glutamine. Kasumi-1 cells were maintained at a cell density between 3 × 10^5^ and 6 × 10^5^ cells/mL, and split at a ratio of about 1:2 to 1:3 every 3 to 4 days to ensure adequate cell viability.

### Cell viability assays

Aliquots of cells were treated with each inhibitor at concentrations ranging from 4 nM to 20 µM or with a combination of inhibitors (with fixed ratio) for 48 h (in media, as above), whereas the control group was treated with vehicle [DMSO, 0.1 % (vol/vol)]. The MTS reagent was added to the cells afterwards. The resulting fluorescent signals were measured after one to four hours using a fluorescence plate reader. Viability was calculated as a percentage of cells treated with DMSO. Numerical IC50 values were calculated by nonlinear best-fit regression analysis using the Prism 8 software (GraphPad, Inc.).

### Analysis of synergistic effects

Synergy/antagonism between drugs was estimated from the combination index (CI). The calculation of CI was performed with the CalcuSyn program (BioSoft, Cambridge, UK) that implements the algorithm of Chou-Talalay [32]. To perform this statistical analysis, we employed the data from cell viability and proliferation assays described above. CI values were generated from a range of growth inhibition (fraction affected, Fa) levels, from 0.1 to 0.9. CI < 1, CI = 1, and CI > 1 represent synergism, additivity, and antagonism, respectively [33].

### Measurements of cell growth rates with drug combinations

To explore the effects of combining different inhibitors, AML cells lines were incubated with palbociclib and PI3K inhibitor at concentrations that match the IC30 values of each inhibitor (Tables 1 and 2). The cells which were treated with blank medium with the same DMSO concentration (0.1 %) as the experiment were set as control. The experiments were run in 24-well plates. Each well was set up with 1 × 10^5^ cells in 1 mL medium with the indicated inhibitors. Briefly, cells were allowed to grow for four days, after which they were washed, reseeded and allowed to grow again for a total of four generations. The concentrations of the cells were measured every two days by noninvasive counting, and the growth rates were estimated by using the using the ratrack tool [34] https://github.com/Sandalmoth/ratrack.

**Table 1:** Concentrations of inhibitors, which match their IC30 values, as used for the combination treatment of AML cells

	Palbociclib (µM)	Quizartinib (nM)
MOLM-13	0.05	0.37
MOLM-14	0.58	0.21
MV4-11	0.61	0.11

**Table 2:** Concentrations of inhibitors (µM) for the combination treatment to AML cells

	Palbociclib	Idelalisib	Duvelisib	Copanlisib	Alpelisib
MOLM-13	0.05	8.00	0.14	0.03	0.30
MOLM-14	0.58	2.90	0.15	0.02	1.36
MV4-11	0.61	12.67	0.06	0.03	0.10

### Preparation of protein extracts and immunoblotting

MOLM-14 and MV4-11 were treated with DMSO [control group, 0.1% (vol/vol)], palbociclib (0.02 or 0.1µM), quizartinib (0.1 or 1 nM), copanlisib (0.1 or 0.5 µM) and combinations of palbociclib with the other inhibitors for 24 h. After centrifugation at 300×*g*, the cell pellets were washed by ice-cold Phosphate Buffered Saline (PBS), suspended, and lysed in cell lysis buffer (25 mM Tris–HCl at pH 7.6, 150 mM NaCl, 1% NP-40, 1% sodium deoxycholate, 0.1% SDS, and protease inhibitor). The protease inhibitor was used according to the manufacturer’s instruction, 1 tablet per 10 mL of extract. After centrifugation at 15,000×*g*, supernatants were used as cell lysates. All lysates were incubated for 5 min at 95 °C and centrifuged for 1 min at 4 °C before determining protein concentrations by a BCA assay.

The proteins were resolved by electrophoresis using 8–16% Tris-Glycine mini protein gel (Invitrogen, Fisher Scientific Sweden) and then transferred to a PVDF membrane (Bio-Rad). For wet immunoblotting, standard procedures were used. The membrane was blocked in TBST buffer containing 5% low fat milk powder for 1 h at room temperature and then incubated overnight with CDK4 (D9G3E) Rabbit mAb (1:1000, Cell Signaling Technology) in TBST buffer containing 5% BSA at 4 °C with gentle shacking. After three washes for 15 min with TBST at room temperature, the membrane was incubated for an additional hour at room temperature with a secondary antibodyMouse Anti-rabbit IgG (conformation specific L27A9) mAb (HRP Conjugate) (1:2000, Cell Signaling Technology) in TBST buffer containing 5% low fat milk powder. To explore the effect of inhibitors on the expression of Phospho-Akt (pSer473), after blocking the membrane for 1 h at room temperature, we incubated the membrane overnight at 4 °C with gentle shacking with Phospho-Akt (Ser473) (193H12) Rabbit mAb (1:1000, Cell Signaling Technology) in TBST buffer containing 5% BSA. After three times wash for 15 min at room temperature, the membrane was incubated with a secondary antibody Goat anti-Rabbit IgG (H+L) HRP conjugate at dilutions 1:20,000 (Thermo Fisher Scientific) for another 1 h in TBST buffer containing 5% low fat milk powder. Immunoreactive bands were detected using Amersham ECL Prime Western Blotting Detection Reagent (Fisher Scientific, Sweden).

### Immunofluorescence microscopy

For immunofluorescence microscopy, MOLM-14 and MV4-11 cells were incubated with quizartinib and palbociclib under the same conditions as in section above. Following preparation and incubation with the drugs, the cells were harvested and washed twice with ice cold PBS buffer. The cells were thereafter fixed by 4% formaldehyde for 20 min at 37 °C and rinsed briefly two times with PBS to remove traces of the fixative. The cells were then smeared on gelatin-coated slides gently with the side of a pipette tip. When the liquid had been evaporated, cells were washed two times with washing buffer (0.1% BSA in PBS). The cells were thereafter blocked in PBS containing 10% normal donkey serum and 0.3% Triton X-100 for 45 min to 1 h at room temperature followed by permeabilisation with PBS containing 0.5 % Tween-20 for 10 min at room temperature. After removing the blocking buffer, cells were incubated with diluted (1:800) primary antibody CDK4 (D9G3E) rabbit mAb in TBST buffer containing 5 % BSA at 4 °C overnight. This was followed by two washes with washing buffer. The preparation continued with the cells incubated with diluted (1:2000) fluorescein-labelled secondary antibody anti-rabbit IgG (H+L), F(ab′)2 Fragment (Alexa Fluor 555 Conjugate, Cell Signaling Technology) in TBST containing 5% low fat milk powder for 1 h at room temperature protected from light. Finally, the cells were rinsed two times with washing buffer, and then counter-stained with 0.1 µg/mL DAPI for nucleic acid staining in the dark. Prior to incubation, the cells were washed two times with PBS, then covered with cover slides by mounting medium.

To quantify the fluorescence of different groups, FIJI software was used. In brief, all images with the same exposure time and image depth were collected and analysed with FIJI. Thereafter, the control group was selected to auto-adjust brightness/contrast since this group had good fluorescence. After this setup, the “Propagate to all other open images” option was used to make all images within the same visual range. For each image, three cells were used to measure the mean intensity.

## Results and discussion

### Palbociclib and PI3K inhibitors effectively hamper the growth of AML cells, with IC50 values in the µM range or lower

To examine whether PI3K and CDK4/6 inhibitors are effective against FLT3^+^-cell lines, the in vitro activity of the inhibitors palbociclib, idelalisib, duvelisib, alpelisib and copanlisib against three AML cell lines were assessed by cell viability assay (Additional file 1: Figs. S1–S5) and the results were used for determination of the inhibitors’ IC50 (Table 3). All agents were effective in hampering the growth of AML cell lines, with IC50 values that ranged from $\sim$20 µM (idelalisib) to less than 100 nM (copanlisib). Palbociclib reduced the viability of MOLM-13, MOLM-14 and MV4-11, with IC50 values of 0.2, 1.9 and 1.1 µM respectively (Table 3). Apparently, the efficacy of the CDK4/6 inhibitor was lower for the cell lines that were homozygous for FLT3-ITD (MOLM-14 and MV4-11 are homozygous, MOLM-13 is heterozygous). In contrast with palbociclib, the efficacy of PI3K inhibitors against the tested AML cell lines does not seem to depend on the FLT3 mutation status and is similar (mostly within 50% of the IC50 values) between the cell lines. Idelalisib, which is a selective PI3K$\delta$ inhibitor, had the lowest efficacy against all cell lines. Interestingly, idelalisib is used to treat another type of leukaemia, chronic lymphocytic leukaemia (CLL), where it targets cell survival and proliferation [35], but does not necessarily induce apoptosis. In our assay that uses cell counts, its effects on AML cell appears modest. Alpelisib, a selective PI3K$\alpha$ inhibitor, was about one order of magnitude more effective than idelalisib but less efficacious than the other PI3K inhibitors. Duvelisib, which is a PI3K$\gamma \delta$ inhibitor, inhibited cell growth with IC50 values that were about 1 µM or less. Finally, copanlisib, a pan-PI3K inhibitor had the best effect in terms of the IC50. Overall, the results show that PI3K inhibitors with activity against multiple isoforms of PI3K are more effective than the more selective inhibitors. In particular, inhibition of PI3K$\delta$ was not enough to significantly inhibit AML growth unless concentrations higher than 10 µM were used.

**Table 3:** IC50 values of AML cell lines in the presence of inhibitors

	Idelalisib (µM)	Duvelisib (µM)	Alpelisib (µM)	Copanlisib (nM)	Palbociclib (µM)
MOLM-13	14.9 ± 0.03	0.4 ± 0.06	1.2 ± 0.05	89.1 ± 0.05	0.2 ± 0.06
MOLM-14	12.5 ± 0.1	1.3 ± 0.1	2.9 ± 0.03	66.5 ± 0.05	1.9 ± 0.08
MV4-11	21.1 ± 0.05	0.3 ± 0.14	0.6 ± 0.09	128.8 ± 0.05	1.1 ± 0.03

Numerical IC50 values were calculated with non-linear best-fit regression analysis using the Prism 8 software. See Additional file 1: Figs. S1–S5 for concentration-dependent normalised cell viabilities

### Palbociclib combined with quizartinib displays partially synergistic inhibition against leukaemic cells

**Fig. 1:**
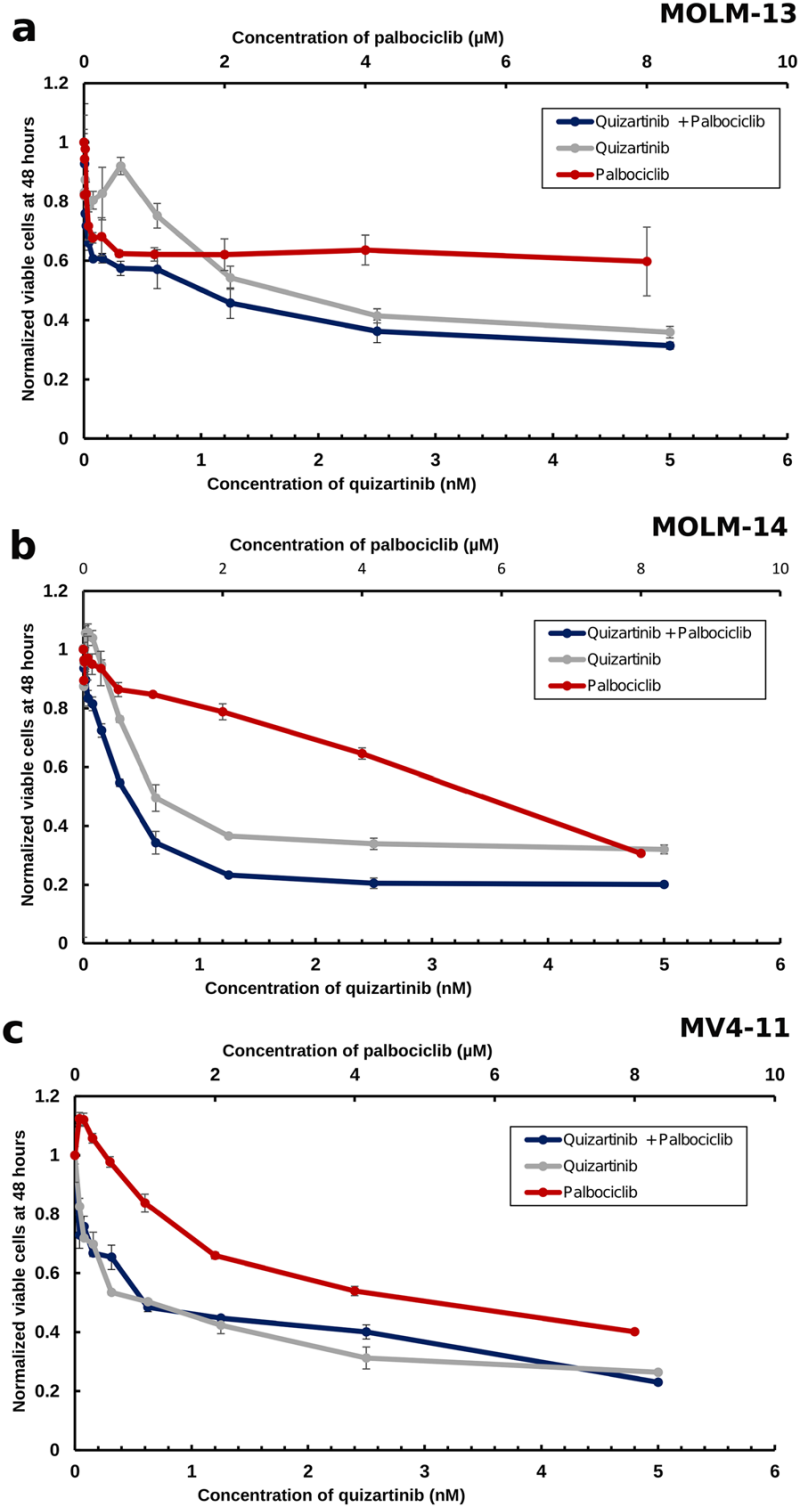
Normalised cell viabilities of MOLM-13 (**a**), MOLM-14 (**b**) and MV4-11 (**c**) cells after 48 h treated with palbociclib (red lines), quizartinib (grey lines) and a combination between them (dark blue lines). In all subfigures, the x-axis on the bottom showed the concentration of quizartinib while the top axis shows the concentration of palbociclib. Error bars represent standard deviations calculated from three measurements

After studying the CDK4/6 and PI3K inhibitors individually, we investigated whether the combination of palbociclib with quizartinib could inhibit cell proliferation synergistically in AML cells. To this end, AML cell lines were treated with these inhibitors for 48 h at different concentrations (Fig. 1). The addition of palbociclib to quizartinib caused a dose-dependent decrease in cell proliferation. The calculated combination index (CI) values in this experiment (Table 4) confirmed that the effect of co-treatment with palbociclib and quizartinib is synergistic as long as the combined effect on growth is up to 50% (fraction affected, Fa ≤ 0.5 in all cell lines) and overall in MOLM-14 cells. Fa is the percent of cells affected by the therapy, i.e., Fa = 0.5 corresponds to limiting the growth by 50%.

**Table 4:** Combination index for palbociclib combined with PI3K inhibitors against three different AML cell lines

Cell Line	Inhibitor (+palbociclib)	Combination index			
		Fa 0.25	Fa 0.5	Fa 0.75	Fa 0.9
MOLM-13	Copanlisib	0.03	0.20	1.16	7.21
MOLM-13	Alpelisib	0.64	0.86	1.18	1.63
MOLM-13	Duvelisib	0.06	0.27	1.24	5.78
MOLM-13	Idelalisib	0.73	0.87	1.05	1.28
MOLM-13	Quizartinib	0.06	0.54	5.02	46
MOLM-14	Copanlisib	0.32	0.44	0.61	0.84
MOLM-14	Alpelisib	0.39	0.30	0.23	0.17
MOLM-14	Duvelisib	0.25	0.19	0.25	0.34
MOLM-14	Idelalisib	0.71	0.27	0.18	0.15
MOLM-14	Quizartinib	0.05	0.02	0.01	0.006
MV4-11	Copanlisib	0.10	0.32	1.07	3.96
MV4-11	Alpelisib	0.84	0.71	0.62	0.56
MV4-11	Duvelisib	0.24	0.95	3.79	15
MV4-11	Idelalisib	0.11	0.34	1.43	6.56
MV4-11	Quizartinib	0.05	0.28	1.79	11.27

CIs were calculated with Calcusyn

### A combination of low dose palbociclib and quizartinib effectively inhibits cell growth and does not lead to resistance

Quizartinib is a highly specific FLT3 inhibitor which inhibited the growth of MOLM-13, MOLM-14 and MV4-11 cells with IC50 values 0.62 ± 0.03, 0.38 ± 0.06 and 0.31 ± 0.05 nM (Additional file 1: Fig. S6). However, resistance to quizartinib is developed quickly in patients, and experiments in AML cell lines corroborated this [23]. Using a protocol to follow on the development of resistance in cell lines, we have studied how the cells reacted to successive treatments with quizartinib, palbociclib, or a combination thereof. The drugs were used in concentrations that match their IC30 values (Table 1).

**Fig. 2:**
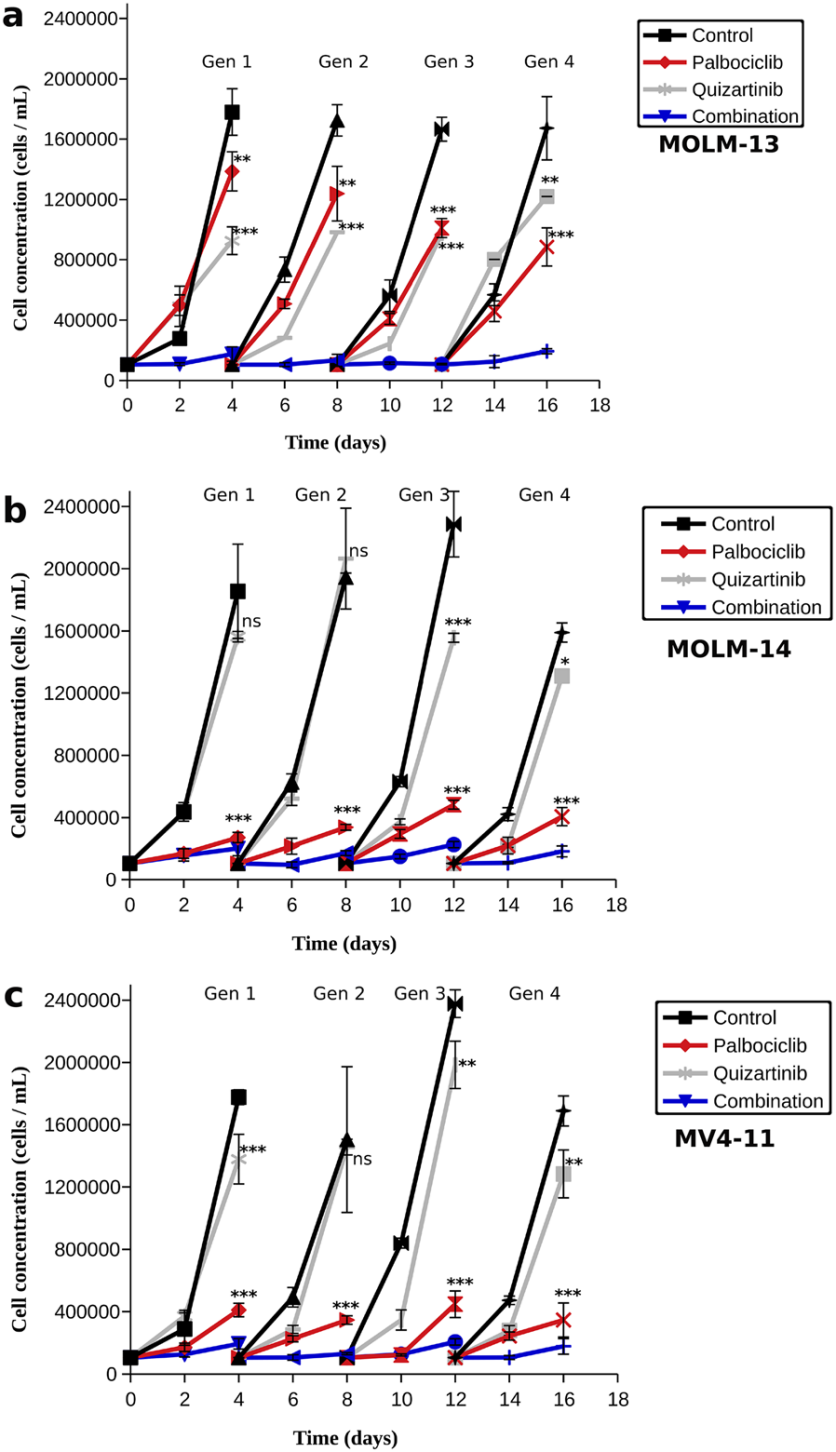
Cell growth of AML cell lines treated by quizartinib, palbociclib, or a combination thereof. Error bars are calculated from four experiments **a** MOLM-13 cells, **b** MOLM-14 cells, **c** MV4-11 cells. ANOVA with Tukey’s post hoc test—one asterisk indicates p < 0.05 between experiment and control, two asterisks indicate p < 0.01, three asterisks indicate p < 0.001 while ns means p > 0.05

As shown in Fig. 2, both drugs were initially effective against the AML cell lines, but cell growth became much faster after 48 h treatment with quizartinib. Palbociclib remained effective throughout the therapy, but its efficacy was lower to begin with. Interestingly, the efficacy of palbociclib increased with the generation in MOLM-13 cells. The combinatorial strategy (blue lines) markedly decreased cell growth in all cell lines. Moreover, no signs of resistance to combination therapy were observed. Thus, our results suggest that the combination strategy involving CDK4/6 inhibitors and quizartinib might overcome resistance with lower doses of the inhibitors.

### Palbociclib and quizartinib reduce the expression of FLT3 on the cell surface

**Fig. 3:**
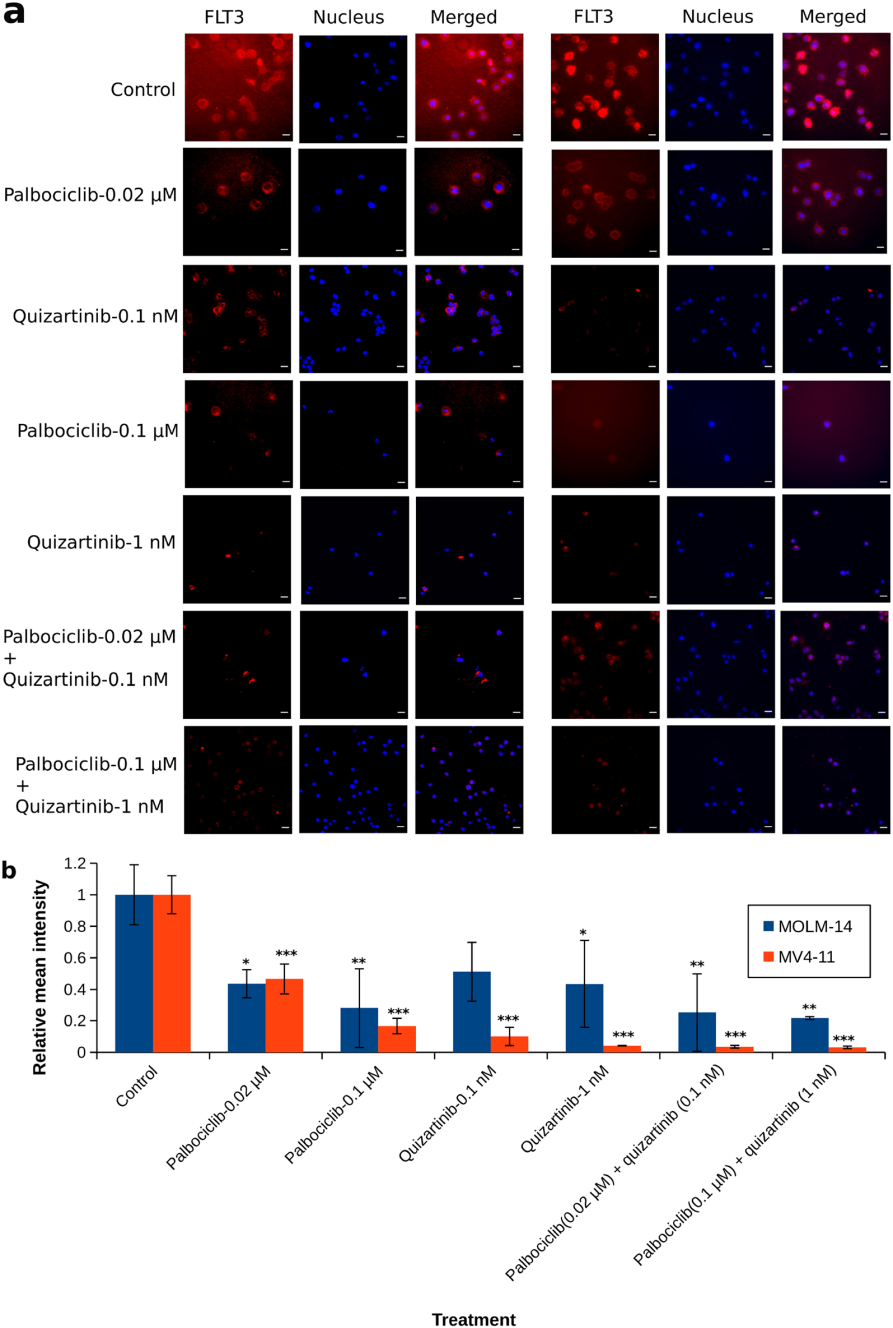
Confocal imaging to assess the expression of FLT3 in MOLM-14 (**a**, left panel) and MV4-11 (**a**, right panel) cell lines. The cells were treated with different drug protocols as indicated. Immunofluorescence analysis was performed using an antibody against FLT3. In both panels: FLT3, FLT3 antibody staining (red signal); Nucleus, DAPI nucleus staining (blue signal); Merged, merged image of FLT3 and DAPI. Scale bar: 200 µm. **b** ANOVA with Tukey’s post hoc test, based on calculated mean intensity of FLT3 fluorescence. One asterisk indicates p < 0.05 between experiment and control, two asterisks indicate p < 0.01 while three asterisks indicate p < 0.001

To better understand the mechanism by which the cells are inhibited when palbociclib alone or in combination with quizartinib inhibit cellular growth, we examined the suppression of FLT3 expression in the nucleus of cancer cells and its migration to the cell membranes (where it acts as a receptor tyrosine kinase) by immunofluorescence staining on AML cell lines. Palbociclib and quizartinib interfered with the expression of FLT3 on the cell surface in a concentration-dependent fashion (Fig. 3a). As a result, palbociclib and quizartinib act cooperatively to inhibit FLT3-dependent signalling. What is more, the effect on MV4-11 cells was more pronounced when compared with MOLM-14 (Fig. 3b). Interestingly, palbociclib at the experimental concentrations and incubation time had limited effect on FLT3 phosphorylation (Additional file 1: Fig. S7), which is in line with a previous study [12]. Thus, the cells react to simultaneous application of palbociclib and quizartinib by reducing the expression of FLT3, but it still gets phsphorylated even when CDK4 and CDK6 are inhibited. All together, the results are in line with a prediction, based on the interaction network of AML [36], which suggested that the combination between FLT3 and CDK4/6 inhibitors would have a combined effect on FLT3. CDK6 is an upstream regulator of FLT3 and its inhibition reduces the expression of the latter [12, 15].

### CDK4 is expressed in AML cell lines, and its expression is reduced by a combination of palbociclib and quizartinib

**Fig. 4:**
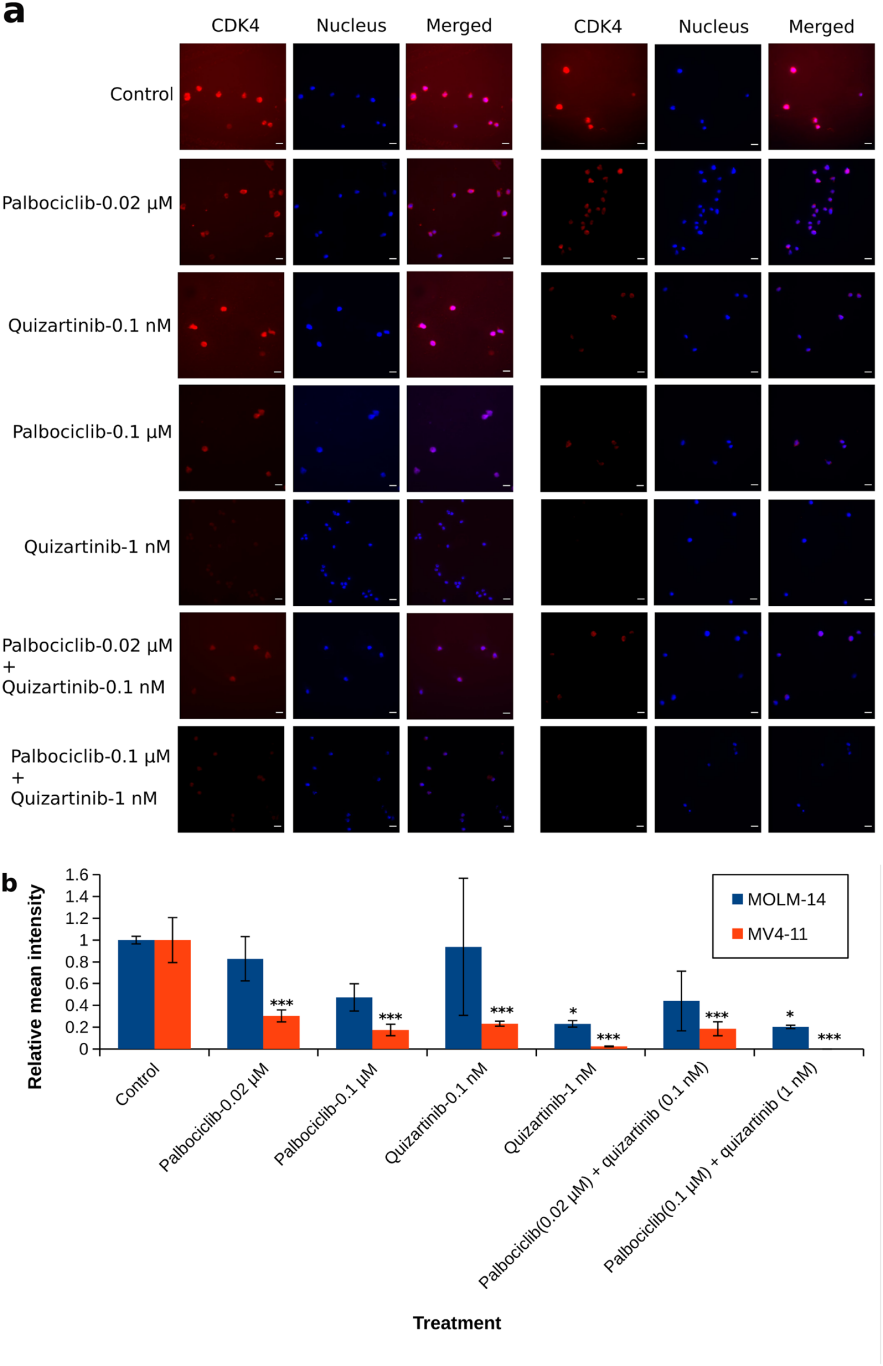
Confocal imaging to assess the expression of CDK4 in MOLM-14 (**a**, left panel) and MV4-11 (**a**, right panel) cell lines. The cells were treated with different drug protocols as indicated. Immunofluorescence analysis was performed using an antibody against CDK4. In both panels: CDK4, CDK4 antibody staining (red signal); Nucleus, DAPI nucleus staining (blue signal); Merged, merged image of CDK4 and DAPI. Scale bar 200 µm. **b** ANOVA with Tukey’s post hoc test, based on calculated mean intensity of CDK4 fluorescence. One asterisk indicates p < 0.05 between experiment and control, two asterisks indicate p < 0.01 while three asterisks indicate p < 0.001

Since both palbociclib and quizartinib inhibit cell growth, and as combining these inhibitors limited the expression of FLT3 on the cell surface, we next analysed whether this combination had a similar effect on CDK4 expression in AML cells. Palbociclib inhibits both CDK4 and CDK6. Hitherto, only CDK6 was identified as a key player in AML [12, 15, 17, 37, 38], and we sought to examine if CDK4 might also play a role in signalling in AML cells. Thus, we studied the expression of CDK4 and explored the influence of combination protocols on CDK4 signalling. Indeed, CDK4 was expressed in the cells (Fig. 4a). After exposing cells to increasing concentrations of palbociclib, immunofluorescent staining analysis and calculated mean intensities showed dose-dependent declines in the levels of CDK4 in MV4-11 cells. Of note, quizartinib exhibited a pronounced inhibition on CDK4 expression in AML cell lines (Fig. 4b). Quizartinib in high concentration completely blocked CDK4, as observed by western blot analysis (Additional file 1: Fig. S8). Together, our measurements show that CDK4 is widely expressed in AML cells, and that the cells reacted to a combination of palbociclib and quizartinib by lowering FLT3 and CDK4 expression. While FLT3 expression is likely inhibited by blocking CDK6, it is not known why the expression of CDK4 is inhibited. There might be positive feedback between FLT3 and CDK4, so that blocking the former reduces the expression of the latter. This might explain how this combination can overcome resistance to treatments in AML cells.

### A combination of palbociclib and quizartinib inhibits the phosphorylation of Akt in AML cells

The PI3K/Akt pathway plays an important role in preventing cells from undergoing apoptosis. For instance, activated Akt is involved in a great variety of biological processes, such as cell proliferation [39]. Furthermore, in AML, Akt is affected by CDK6 [15]. To study the influence of combination protocols on the expression of p-Akt in AML cells, we examined the presence of p-Akt (phosphorylated at Ser^473^) by western blotting (Additional file 1: Fig. S9). The results suggest that the indicated inhibitors carry a dose-dependent inhibition of the expression of p-Akt.

### Palbociclib combined with PI3K inhibitors displays anti-proliferative activity against leukaemic cells

**Fig. 5:**
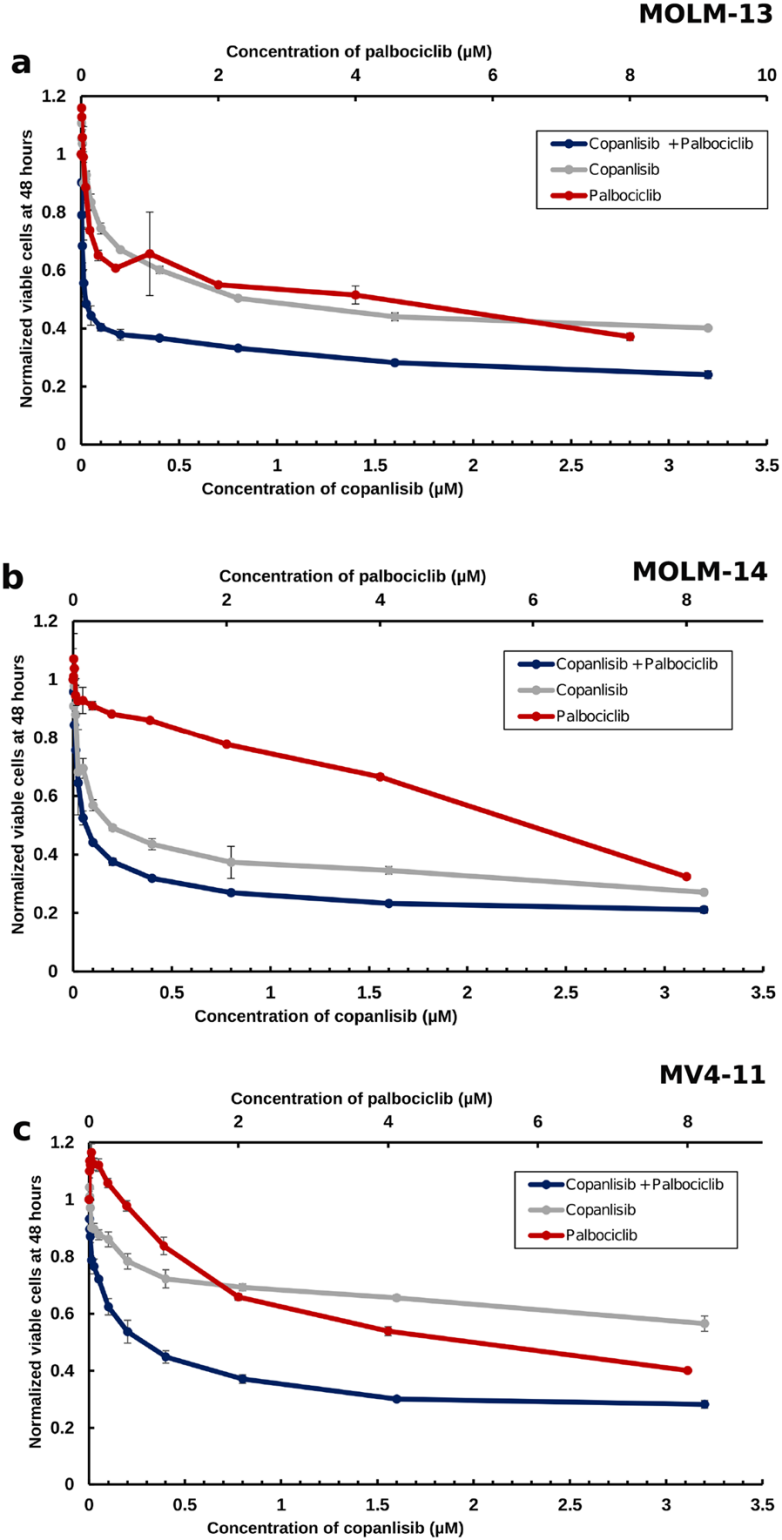
Normalised cell viabilities of MOLM-13 (**a**), MOLM-14 (**b**) and MV4-11 (**c**) cells after 48 h of treatment with copanlisib (grey lines). Dark blue lines in all subfigures represented the combination of palbociclib with the copanlisib and red lines represent cells treated with palbociclib. In all subfigures, the x-axis on the bottom shows the concentration of the PI3K inhibitor while the x-axis on the top shows the concentration of palbociclib. Error bars represent standard deviations calculated from three measurements

Given that palbociclib and PI3K inhibitors inhibited the growth of AML cells, and that palbociclib could be combined with quizartinib for greater effect against tumour growth, we decided to investigate the effect of palbociclib and PI3K inhibitors in combination on these cells. A previous study suggested that such combination might be effective based on a computer aided analysis [36]. To this end, AML cell lines were treated for 48 h with these inhibitors. The addition of palbociclib to PI3K inhibitors (Fig. 5 and Additional file 1: Fig. S22) caused a dose-dependent decrease in cell proliferation. In order to examine whether the effect of the co-treatment with PI3K and palbociclib is synergistic, we calculated the combination index (CI) values by varying the concentrations of palbociclib in this experiment from 0.004 to 8 µM (keeping the ratio of palbociclib to PI3K inhibitor fixed for each individual inhibitor), as shown in Table 4 and Additional file 1: Figs. S10–S13. Synergism was observed with all cell lines and treatments as long as the affected growth was ≤ 50%. As with palbociclib and quizartinib, MOLM-14 cells were more sensitive to combination therapy and in those cells and synergism was observed throughout whole range of Fa values.

### Palbociclib in combination with PI3K inhibitors overcomes resistance to therapy

PI3K inhibitors inhibited the proliferation of AML cells after 48 h treatment (Additional file 1: Figs. S2–S5). However, longer exposure to the drugs and following on several generations of AML cells revealed drug resistance to PI3K inhibitors (Additional file 1: Fig. S23, grey lines; note the increase in growth rates after 48 h in almost all experiments).

Combinations of palbociclib and a PI3K inhibitor (blue lines in Additional file 1: Fig. S23) significantly suppressed cell growth in all cell lines. More importantly, no signs of drug resistance were observed to the combination therapy. The cell growth rates on day 2 of every generation were calculated, revealing that the combined treatment was associated with a greater decrease in growth rate, in comparison with single drug treatments (Additional file 1: Tables S1–S3). These results indicated that together with PI3K inhibitors, palbociclib inhibited the cell growth for a longer duration, and suggested that such a combination might be an effective strategy to overcome drug resistance.

### Copanlisib regulated the expression of CDK4 and p-Akt

Considering the fact that for many tumour cells, inhibition of CDK4/6 can induce cellular quiescence or senescence [40–43], we evaluated whether CDK4 expression was affected by copanlisib alone or in combination with palbociclib. Copanlisib was selected as it was more effective than other PI3K inhibitors on its own. While the cells did not react to palbociclib by reducing the expression of CDK4, copanlisib lead to dose-dependent downregulation in CDK4 expression, especially when combined with palbociclib (Additional file 1: Fig. S8). Moreover, the cells did not express p-Akt following treatment with copanlisib (Additional file 1: Fig. S9).

### The efficacy of gilteritinib and abemaciclib against AML cells

Given that quizartinib and palbociclib were effective together against AML cell growth, we further tested two newer FLT3 and CDK4/6 inhibitors, namely gilteritinib and abemaciclib. The in vitro activities of these inhibitors were studied (Additional file 1: Figs. S14, S15) and their IC50 values were determined (Table 5). The measured values were similar to those obtained for quizartinib (Additional file 1: Fig. S6) and palbociclib (Table 3).

**Table 5:** IC50 values of gilteritinib and abemaciclib measured in AML cell lines

	Gilteritinib (nM)	Abemaciclib (µM)
MOLM-13	0.99 ± 0.14	0.53 ± 0.18
MOLM-14	3.60 ± 0.04	0.24 ± 0.17
MV4-11	1.85 ± 0.06	3.83 ± 0.09

Numerical IC50 values were calculated with non-linear best-fit regression analysis using the Prism 8 software. See Additional file 1: Figs. S12 and S13 for concentration-dependent normalised cell viabilities

### Gilteritinib displays synergistic inhibition against leukaemic cells when combined with palbociclib or copanlisib, but weaker synergy with abemaciclib

To study the effect of combining gilteritinib with palbociclib, abemaciclib or copanlisib against AML cells, we incubated AML cells with these inhibitors for 48 h at different concentrations (Additional file 1: Fig. S24). The resulting CI values were < 1 over most of the fraction affected when gilteritinib was combined with palbociclib or copanlisib, but not with abemaciclib (Table  6, Additional file 1: Figs. S17, S19 and S20). Apparently, gilteritinib had a stronger synergistic effect than quizartinib when combined with either palbociclib or copanlisib. Abemaciclib is less specific than palbociclib as it also inhibits other CDKs [44] which might explain why combinations with abemaciclib are less synergistic.

**Table 6:** Combination index for gilteritinib combined with PI3K or CDK6 inhibitor against three different AML cell lines

Cell line	Inhibitor (+gilteritinib)	Combination index			
		Fa 0.25	Fa 0.5	Fa 0.75	Fa 0.9
MOLM-13	Palbociclib	0.60	0.73	0.88	1.08
MOLM-13	Copanlisib	0.01	0.07	0.35	1.64
MOLM-13	Abemaciclib	2.25	0.96	0.41	0.18
MOLM-14	Palbociclib	0.03	0.06	0.09	0.16
MOLM-14	Copanlisib	0.80	0.36	0.16	0.07
MOLM-14	Abemaciclib	0.18	0.23	0.28	0.35
MV4-11	Palbociclib	0.04	0.26	1.45	8.15
MV4-11	Copanlisib	0.05	0.05	0.04	0.04
MV4-11	Abemaciclib	2.07	1.69	1.38	1.14

CIs were calculated with Calcusyn

### Gilteritinib combined with CDK4/6 or PI3K inhibitors delays the emergence of resistance

**Fig. 6:**
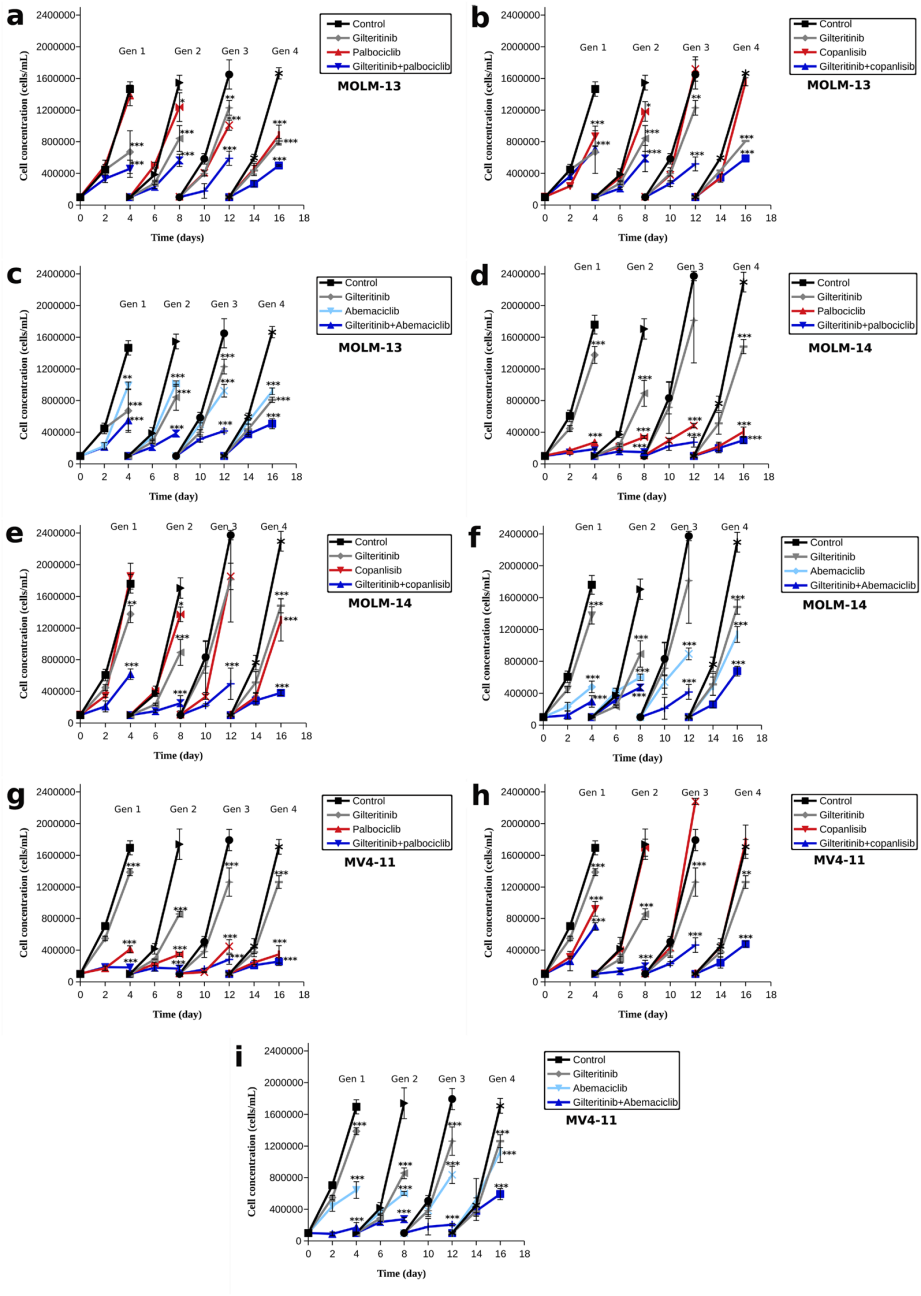
Cell growth of AML cell lines- MOLM-13 (**a**–**c**), MOLM-14 (**d**–**f**) and MV4-11 (**g**–**i**)-treated by gilteritinib, palbociclib (**a**, **d**, **g**) or a combination thereof; gilteritinib, copanlisib (**b**, **e**, **h**) or a combination thereof; gilteritinib, abemaciclib (**c**, **f**, **i**) or a combination thereof. Error bars are calculated from four experiments. ANOVA with Tukey’s post hoc test. One asterisk indicates p < 0.05 between experimental group and control, two asterisks indicate p < 0.01, three asterisks indicate p < 0.001 while ns indicates p > 0.05. The concentrations of the inhibitors match their IC30, Tables  2 and 7

Given that gilteritinib is used in the clinic to treat FLT3^+^-AML, and that resistance is often observed to treatment we set to examine the ability of drug combinations involving gilteritinib to override drug resistance in AML cells, studying it together with palbociclib, abemaciclib or copanlisib, where the concentration of each inhibitor matched its IC30 value (Tables 2 and 7). Continuous, simultaneous exposure of AML cells to combined inhibitors resulted in dramatic decrease of cell numbers relative to monodrug or no treatment in vitro (Fig. 6). Resistance was not observed with the combination therapies, while there are some indications of resistance with monotherapies (more often with copanlisib and abemaciclib). Taken together, these results show that combining gilteritinib with either CDK4/6 or PI3K inhibitors may overcome the drug resistance.

**Table 7:** Concentrations of inhibitors for the combination treatment to AML cells

	Gilteritinib (nM)	Abemaciclib (µM)
MOLM-13	0.26	0.04
MOLM-14	1.67	0.02
MV4-11	1.08	1.53

### Examination of the synergism in combinations involving abemaciclib

While abemaciclib is an effective CDK4/6 inhibitor it is less specific than palbociclib and its combination with gilteritinib was less synergistic (Table 6). It was however effective together with gilteritinib against cell growth and the combination did not lead to resistance in our assay (Fig. 6). Hence, we examined whether combinations of abemaciclib with copanlisib or quizartinib show synergism. The results (Additional file 1: Figs. S16, S18 and S25; Table 8) do not show a general synergistic effect. Interestingly at high inhibition (Fa ≥ 0.5) abemaciclib was synergistic with quizartinib in cells that were homozygous to FLT3-ITD.

**Table 8:** Synergism as measured by combination index for abemaciclib combined with copanlisib or FLT3 quizartinib measured against three different AML cell lines

Cell line	Inhibitor (+abemaciclib)	Combination index			
		Fa 0.25	Fa 0.5	Fa 0.75	Fa 0.9
MOLM-13	Copanlisib	0.15	0.39	1.01	2.64
MOLM-13	Quizartinib	2.19	1.86	1.57	1.34
MOLM-14	Copanlisib	2.85	0.88	0.46	0.31
MOLM-14	Quizartinib	1.37	0.37	0.10	0.04
MV4-11	Copanlisib	0.002	0.21	18	1682
MV4-11	Quizartinib	4.18	0.97	0.22	0.05

CI values were calculated with Calcusyn

### Combinations of abemaciclib with copanlisib or quizartinib limit the emergence of drug resistance

**Fig. 7:**
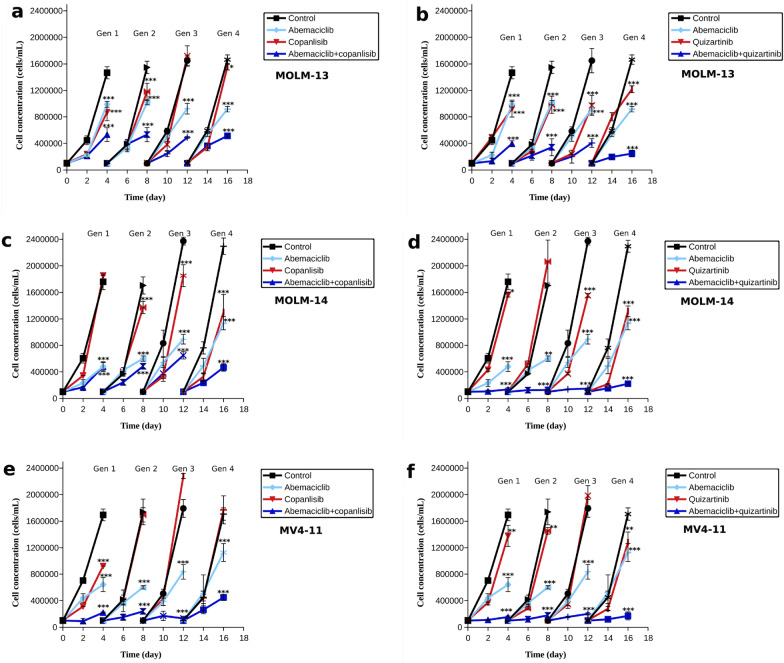
Cell growth of AML cell lines- MOLM-13 (**a**, **b**), MOLM-14 (**c**, **d**) and MV4-11 (**e**, **f**) treated with abemaciclib, copanlisib (**a**, **c**, **e**) or a combination thereof; abemaciclib, quizartinib (**b**, **d**, **f**) or a combination thereof. Error bars were calculated from four experiments. ANOVA with Tukey’s post hoc test: one asterisk indicates p < 0.05 between experimental group and control, two asterisks indicate p < 0.01, three asterisks indicate p < 0.001 while ns indicates p > 0.05. The concentrations of the inhibitors match their IC30 values, Tables 2 and 7

Finally, we set to examine if the combination therapies with abemaciclib can be effective for prevention of drug resistance, when growing the cells with low concentrations of the inhibitors (representing IC30 values). The results show that the addition of abemaciclib to PI3K or FLT3 inhibitor caused a significant decrease of living cell numbers (Fig.  7). Examination of the growth after each generation, resistance was not observed when abemaciclib was combined with quizartinib, and mostly not with companlisib either (there is a slight increase in the number of cells in the fourth generation when MV4-11 cells were treated with abemaciclib and copanlisib, Fig. 7e).

### The efficacy of copanlisib, palbociclib and their combination against wt-FLT3 AML cells

**Fig. 8:**
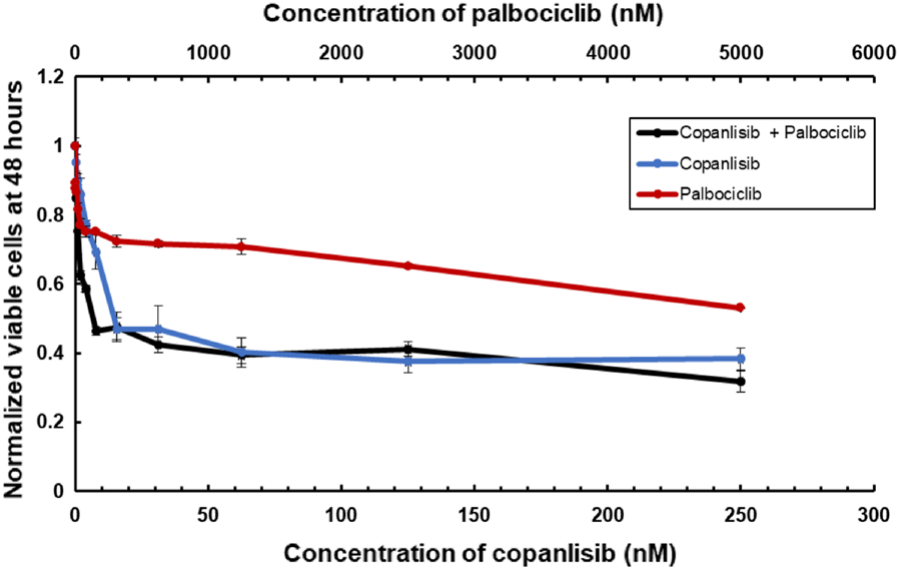
Combinations of copanlisib and palbociclib in Kasumi-1 cells. Normalised cell viabilities of Kasumi-1 cells after 48 h of treatment with copanlisib (blue line), palbociclib (red line) and the combination of two drugs (black line)

Following on the efficacy of combination therapies with PI3K and CDK4/6 inhibitors in FLT^+^-AML cells we wanted to examine the efficacy of such combinations also in an AML cell line that expresses wtFLT3. To this aim, we used Kasumi-1 cells. We first measured the IC50 values for the drugs in Kasumi-1 cells (Table 9). Copanlisib had single digit nM affinity to these cells, which overexpress PI3K. The IC50 value of palbociclib was 2.4 µM, roughly twice higher than for MV4-11 cells. A combination of the drugs was in general as good in inhibiting cell growth as copanlisib alone, which might indicate that CDK4/6 inhibitors are not of much use for such cells (Fig. 8).

**Table 9:** IC50 values of Kasumi-1 cell line in the presence of inhibitors

	Copanlisinb	Palbociclib
Kasumi-1	7.2 ± 0.6 nM	2.4 ± 0.6 µM

## Conclusions

Our study reveals that FLT3^+^-AML cell lines express CDK4, and that quizartinib or copanlisib reduces CDK4 expression. Importantly, given the eventual failure of quizartinib in the clinic owing to drug resistance [8], it is encouraging that the combinations did not show signs for drug resistance. Moreover, the use of a lower dose combination therapy is likely to reduce the risk for side effects. One of the reasons that quizartnib is not in much use today in spite of being highly active is its associated toxicities [45], and a lower dose in combination with another inhibitor might enable more patients to benefit from the drug. Gilteritinib might be better tolerated than quizartinib but drug resistance and toxicities are evident even with gilteritinib, and it is thus encouraging that combination therapies with this agent are effective, at least in cellular models.

Palbociclib is not only an effective combination partner with FLT3 inhibitor, but it acted synergistically with PI3K inhibitors in inhibiting the growth of AML cells. The cells have some sensitivity to palbociclib and pan-PI3K inhibitors, but lower sensitivity to idelalisib. We discovered that combinations of palbociclib or abemacicilib with FLT3 or PI3K inhibitors might overcome resistance to treatment. Here too, cell growth was inhibited with lower inhibitor concentrations, which is expected to reduce toxicities and lead to better tolerance in patients.

Comparing between the different PI3K, CDK4/6 and FLT3 inhibitors that were studied and suggesting the best combinations is challenging. Copanlisib was the most effective as PI3K inhibitor, and is thus most well suited for combination therapy as well. Comparing palbociclib and abemaciclib, the former might act more synergistically with other drugs. Gilteritinib and quizartinib showed overall similar behaviour in this study.

There is much heterogeneity between patients in terms of the genetic composition of tumour cells in AML. This heterogeneity will eventually affect the success of therapy in real world set-up. While we were not able to test the combinations in primary AML cells, it is encouraging that combinations and inhibitors have been shown to be useful in multiple cell lines. It is also encouraging that we did not observe signs of resistance with the combinations. What remains to be seen is whether the combinations of inhibitors would have a sufficient effect when used in low concentrations.

### Supplementary Information

**Additional file 1.** Additional Figures S1–S25, Tables S1–S5.

## Data Availability

This article does not include any generated datasets or non-standard materials.
