# Supplementary material for: Combination strategies to overcome drug resistance in FLT+ acute myeloid leukaemia
Source: Cancer Cell Int. 2023 Aug 11;23:161. doi: 10.1186/s12935-023-03000-x (PMC10416533; doi:10.1186/s12935-023-03000-x)

Combination strategies to overcome drug resistance in FLT$^+$ acute myeloid leukaemia - supplementary information

May 5, 2023

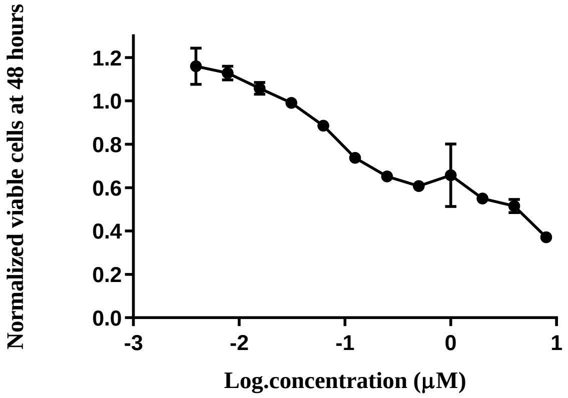

((a)) Normalized cell viability of MOLM-13 after 48 hours in various concentrations of palbociclib

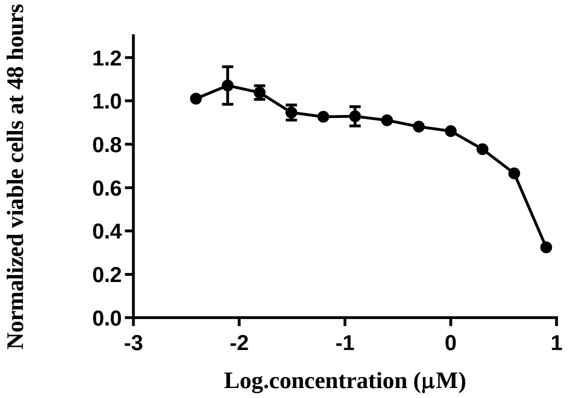

((b)) Normalized cell viability of MOLM-14 after 48 hours in various concentrations of palbociclib

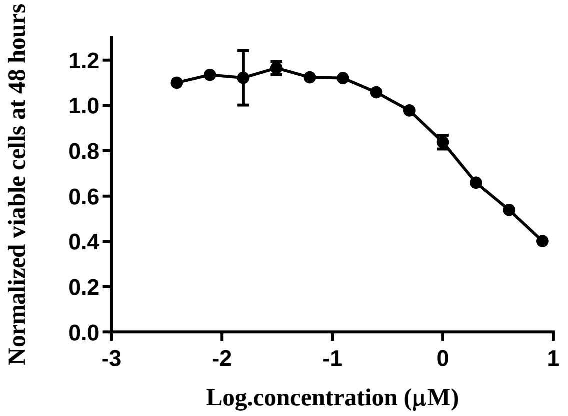

((c)) Normalized cell viability of MV4-11 after 48 hours in various concentrations of palbociclib

Figure S1: Normalized cell viability of AML cell lines after 48 hours in various concentrations of palbociclib

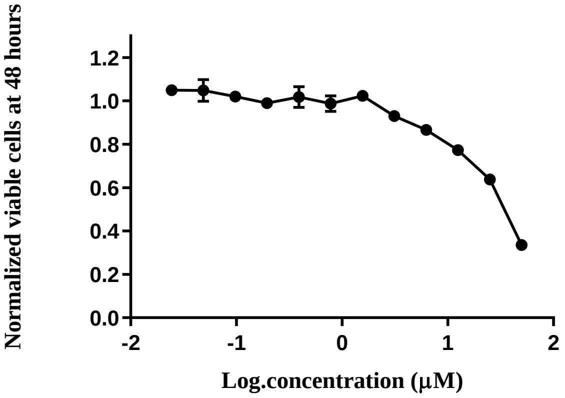

((a)) Normalized cell viability of MOLM-13 after 48 hours in various concentrations of idelalisib

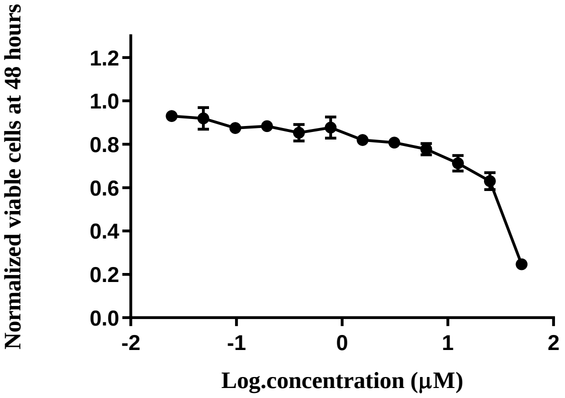

((b)) Normalized cell viability of MOLM-14 after 48 hours in various concentrations of idelalisib

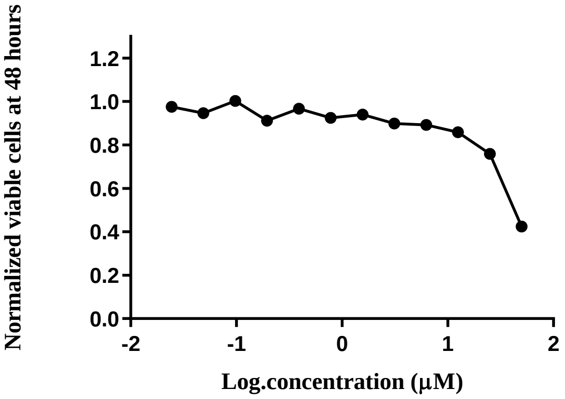

((c)) Normalized cell viability of MV4-11 after 48 hours in various concentrations of idelalisib

Figure S2: Normalized cell viability of AML cell lines after 48 hours in various concentrations of idelalisib

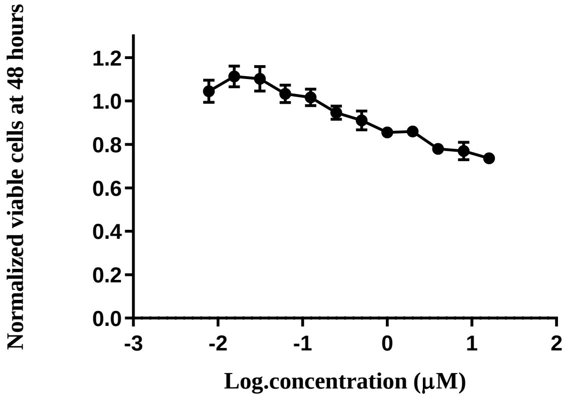

((a)) Normalized cell viability of MOLM-13 after 48 hours in various concentrations of duvelisib

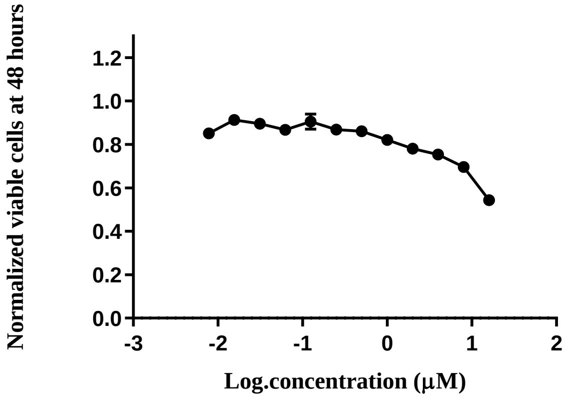

((b)) Normalized cell viability of MOLM-14 after 48 hours in various concentrations of duvelisib

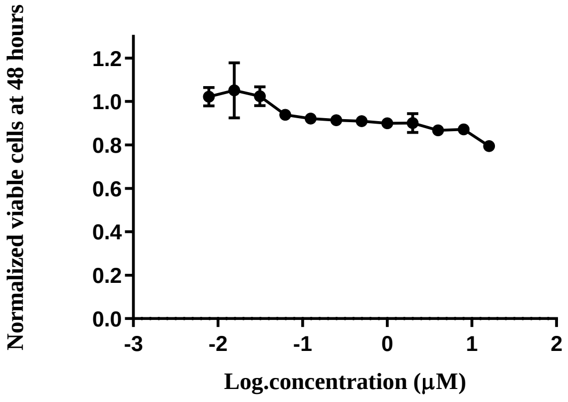

((c)) Normalized cell viability of MV4-11 after 48 hours in various concentrations of duvelisib

Figure S3: Normalized cell viability of AML cell lines after 48 hours in various concentrations of duvelisib

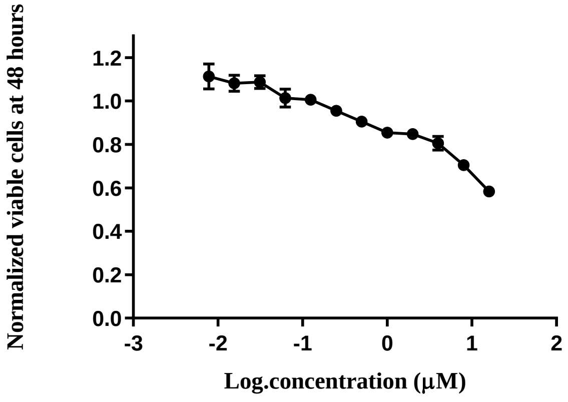

((a)) Normalized cell viability of MOLM-13 after 48 hours in various concentrations of alpelisib

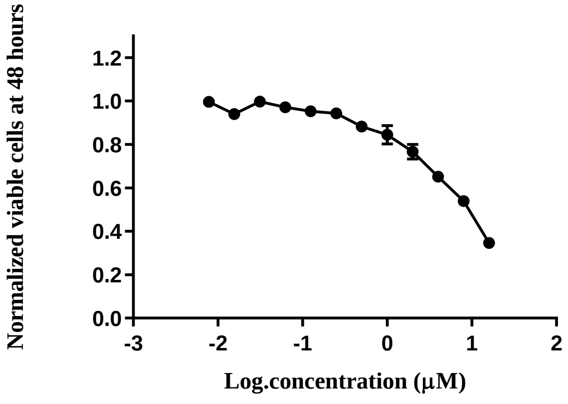

((b)) Normalized cell viability of MOLM-14 after 48 hours in various concentrations of alpelisib

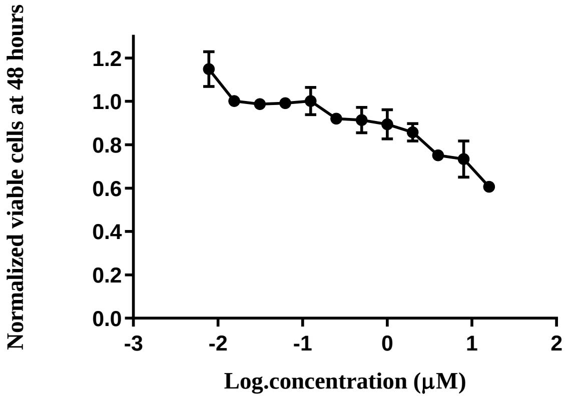

((c)) Normalized cell viability of MV4-11 after 48 hours in various concentrations of alpelisib

Figure S4: Normalized cell viability of AML cell lines after 48 hours in various concentrations of alpelisib

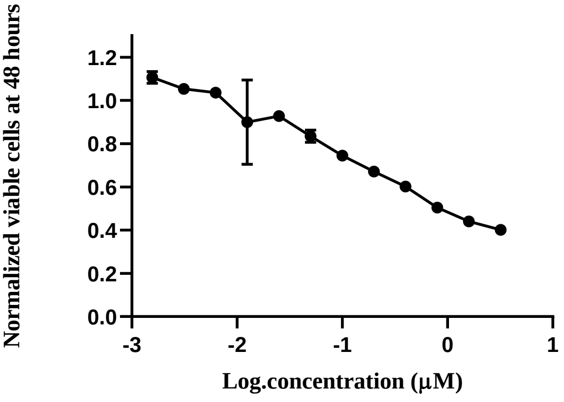

((a)) Normalized cell viability of MOLM-13 after 48 hours in various concentrations of copanlisib

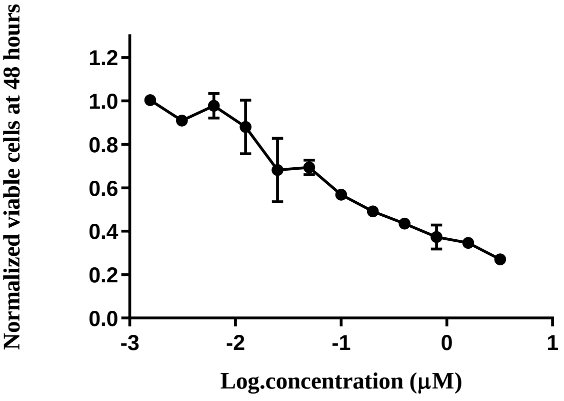

((b)) Normalized cell viability of MOLM-14 after 48 hours in various concentrations of copanlisib

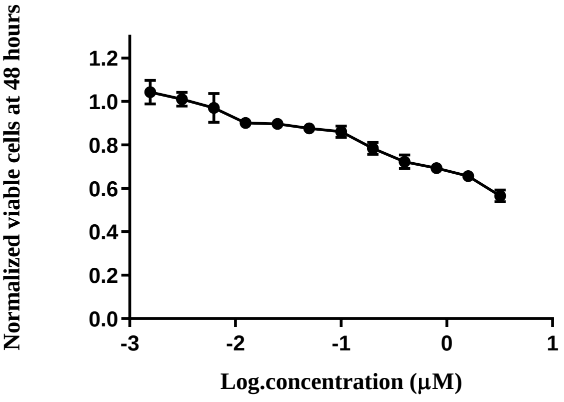

((c)) Normalized cell viability of MV4-11 after 48 hours in various concentrations of copanlisib

Figure S5: Normalized cell viability of AML cell lines after 48 hours in various concentrations of copanlisib

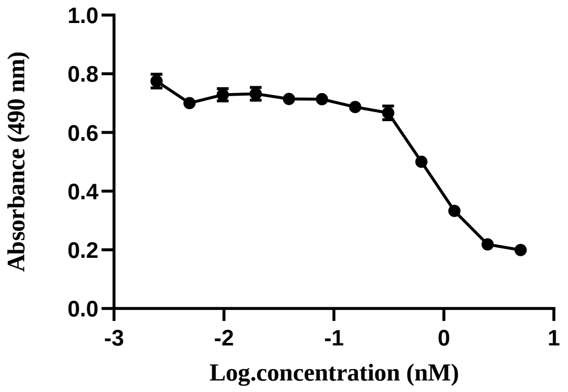

((a)) Normalized cell viability of MOLM-13 after 48 hours in various concentrations of quizartinib

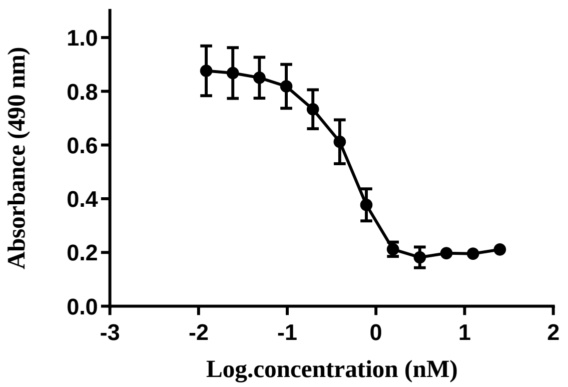

((b)) Normalized cell viability of MOLM-14 after 48 hours in various concentrations of quizartinib

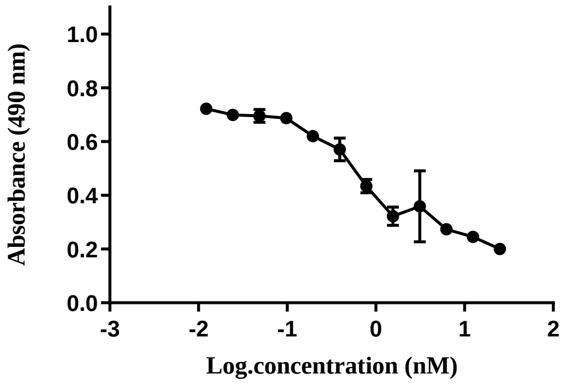

((c)) Normalized cell viability of MV4-11 after 48 hours in various concentrations of quizartinib

Figure S6: Dose-response curve with FLT3 inhibitor quizartinib alone in the AML cell lines. Three independent experiments were carried out.

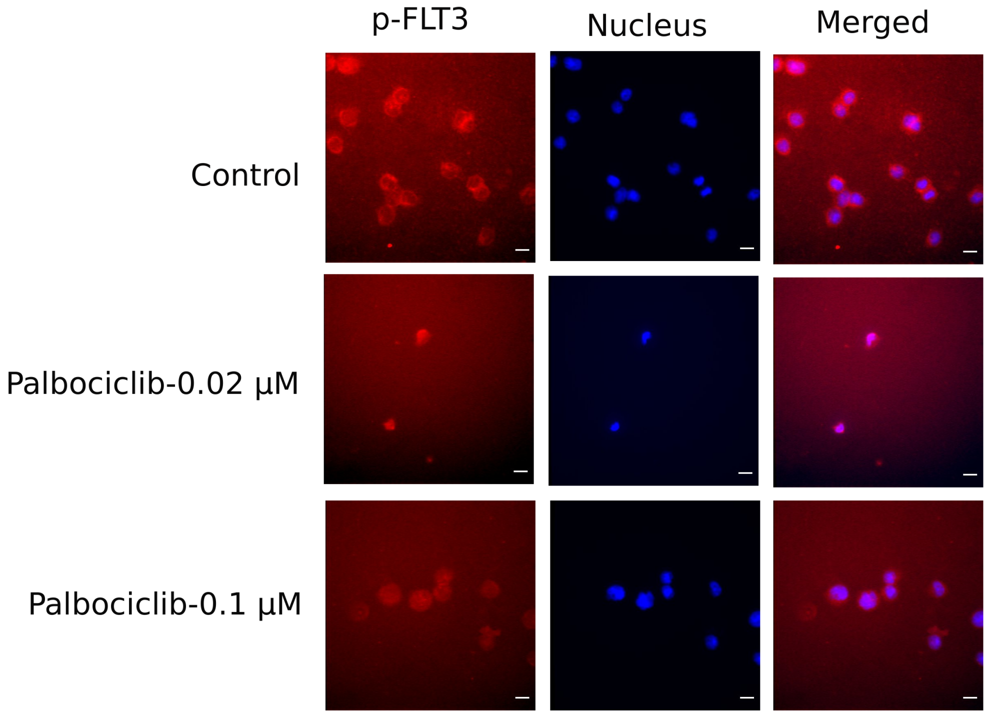

((a)) Staining of phosphorylated FLT3 in MOLM-14 cell line with different concentration of palbociclib treatment after 24 hours

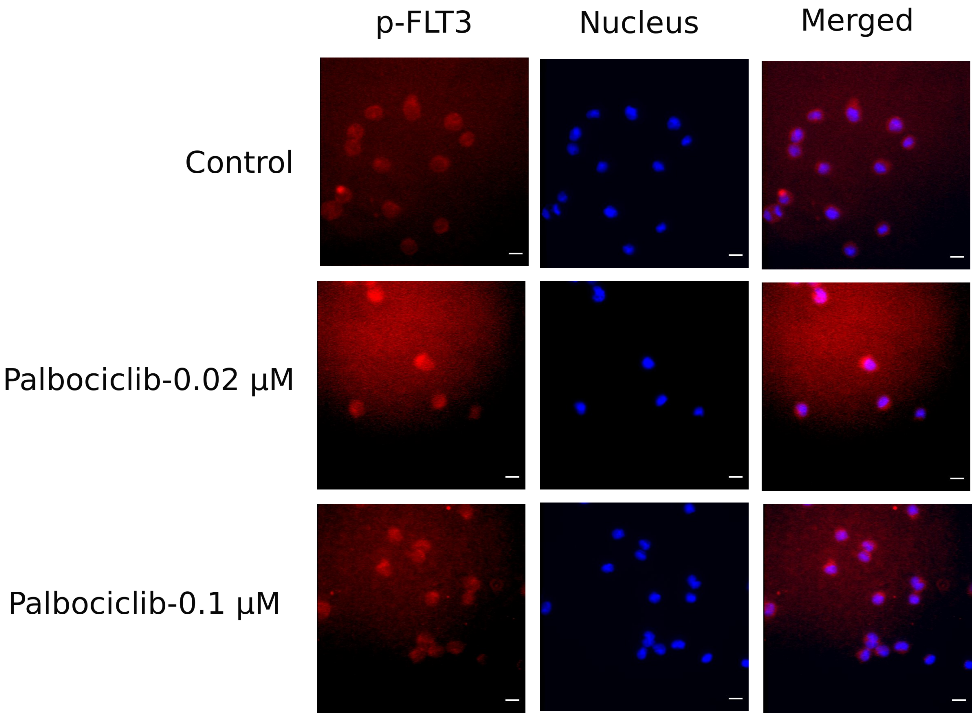

((b)) Staining of phosphorylated FLT3 in MV4-11 cell line with different concentration of palbociclib treatment after 24 hours

Figure S7: Confocal imaging to assess the expression of phosphorylated FLT3 in MOLM-14 (a) and MV4-11 (b) following treatment with palbociclib. Immunofluorescence analysis was performed using an antibody against phosphorylated FLT3. Left panel: phosphorylated FLT3 antibody staining (red signal); Middle panel: DAPI nucleus staining (blue signal); Right panel: merged image. Scale bar 200 μm

Table S 1: Growth rate of MOLM-13 at day 2 under different treatments

**Treatment-generation**	**Growth rate**
**Control-G1**	0.6108
**Control-G2**	0.6879
**Control-G3**	0.7058
**Control-G4**	0.7327
**Palbociclib-G1**	0.7316
**Palbociclib-G2**	0.7017
**Palbociclib-G3**	0.6450
**Palbociclib-G4**	0.4529
**Idelalisib-G1**	0.4566
**Idelalisib-G2**	0.6740
**Idelalisib-G3**	0.8718
**Idelalisib-G4**	0.7294
**Copanlisib-G1**	0.5241
**Copanlisib-G2**	0.5894
**Copanlisib-G3**	0.6723
**Copanlisib-G4**	0.4876
**Alpelisib-G1**	0.4016
**Alpelisib-G2**	0.5163
**Alpelisib-G3**	0.6443
**Alpelisib-G4**	0.7250
**Duvelisib-G1**	0.4729
**Duvelisib-G2**	0.6849
**Duvelisib-G3**	0.6748
**Duvelisib-G4**	0.6029
**Quizartinib-G1**	0.6264
**Quizartinib-G2**	0.6498
**Quizartinib-G3**	0.6476
**Quizartinib-G4**	0.7589
**Idelalisib+Palbociclib-G1**	0.4653
**Idelalisib+Palbociclib-G2**	0.2663
**Idelalisib+Palbociclib-G3**	0.2891
**Idelalisib+Palbociclib-G4**	0.3298
**Copanlisib+Palbociclib-G1**	0.2394
**Copanlisib+Palbociclib-G2**	0.7215
**Copanlisib+Palbociclib-G3**	0.5032
**Copanlisib+Palbociclib-G4**	0.1806
**Alpelisib+Palbociclib-G1**	0.4491
**Alpelisib+Palbociclib-G2**	0.2989
**Alpelisib+Palbociclib-G3**	0.2646
**Alpelisib+Palbociclib-G4**	0.4423
**Duvelisib+Palbociclib-G1**	0.2924
**Duvelisib+Palbociclib-G2**	0.5407
**Duvelisib+Palbociclib-G3**	0.3913
**Duvelisib+Palbociclib-G4**	0.4804
**Quizartinib+Palbociclib-G1**	-
**Quizartinib+Palbociclib-G2**	-
**Quizartinib+Palbociclib-G3**	-
**Quizartinib+Palbociclib-G4**	0.1018

Table S 2: Growth rate of MOLM-14 at day 2 under different treatments

Treatment-generation	Growth rate
**Control-G1**	0.7921
**Control-G2**	0.9372
**Control-G3**	1.0049
**Control-G4**	0.6368
**Palbociclib-G1**	0.2443
**Palbociclib-G2**	0.2430
**Palbociclib-G3**	0.3464
**Palbociclib-G4**	0.3091
**Idelalisib-G1**	0.5824
**Idelalisib-G2**	0.9121
**Idelalisib-G3**	0.6483
**Idelalisib-G4**	0.8395
**Copanlisib-G1**	0.5399
**Copanlisib-G2**	0.7521
**Copanlisib-G3**	0.6730
**Copanlisib-G4**	0.5098
**Alpelisib-G1**	0.4766
**Alpelisib-G2**	0.6316
**Alpelisib-G3**	0.6997
**Alpelisib-G4**	0.5783
**Duvelisib-G1**	0.5681
**Duvelisib-G2**	0.6613
**Duvelisib-G3**	0.8026
**Duvelisib-G4**	0.7726
**Quizartinib-G1**	0.6830
**Quizartinib-G2**	0.9791
**Quizartinib-G3**	0.8501
**Quizartinib-G4**	0.7653
**Idelalisib+Palbociclib-G1**	0.2435
**Idelalisib+Palbociclib-G2**	0.0905
**Idelalisib+Palbociclib-G3**	0.1820
**Idelalisib+Palbociclib-G4**	0.2883
**Copanlisib+Palbociclib-G1**	0.2300
**Copanlisib+Palbociclib-G2**	0.2354
**Copanlisib+Palbociclib-G3**	0.1225
**Copanlisib+Palbociclib-G4**	0.2112
**Alpelisib+Palbociclib-G1**	0.0632
**Alpelisib+Palbociclib-G2**	0.1958
**Alpelisib+Palbociclib-G3**	0.2621
**Alpelisib+Palbociclib-G4**	0.2951
**Duvelisib+Palbociclib-G1**	0.2090
**Duvelisib+Palbociclib-G2**	0.2294
**Duvelisib+Palbociclib-G3**	0.1715
**Duvelisib+Palbociclib-G4**	0.2480
**Quizartinib+Palbociclib-G1**	0.1768
**Quizartinib+Palbociclib-G2**	-
**Quizartinib+Palbociclib-G3**	0.1941
**Quizartinib+Palbociclib-G4**	-

Table S 3: Growth rate of MV4-11 at day 2 under different treatments

Treatment-generation	Growth rate
**Control-G1**	0.6513
**Control-G2**	0.7778
**Control-G3**	1.0971
**Control-G4**	0.6761
**Palbociclib-G1**	0.3541
**Palbociclib-G2**	0.4108
**Palbociclib-G3**	0.7060
**Palbociclib-G4**	0.4992
**Idelalisib-G1**	0.6409
**Idelalisib-G2**	0.8090
**Idelalisib-G3**	0.6802
**Idelalisib-G4**	0.5328
**Copanlisib-G1**	0.6917
**Copanlisib-G2**	0.8024
**Copanlisib-G3**	0.7784
**Copanlisib-G4**	0.6936
**Alpelisib-G1**	0.9883
**Alpelisib-G2**	1.0517
**Alpelisib-G3**	0.5690
**Alpelisib-G4**	0.1978
**Duvelisib-G1**	0.5358
**Duvelisib-G2**	0.2303
**Duvelisib-G3**	0.1895
**Duvelisib-G4**	0.1919
**Quizartinib-G1**	0.6226
**Quizartinib-G2**	0.8228
**Quizartinib-G3**	0.7207
**Quizartinib-G4**	0.5078
**Idelalisib+Palbociclib-G1**	0.2285
**Idelalisib+Palbociclib-G2**	0.2603
**Idelalisib+Palbociclib-G3**	0.3798
**Idelalisib+Palbociclib-G4**	0.1973
**Copanlisib+Palbociclib-G1**	0.1764
**Copanlisib+Palbociclib-G2**	0.2920
**Copanlisib+Palbociclib-G3**	0.3486
**Copanlisib+Palbociclib-G4**	0.2269
**Alpelisib+Palbociclib-G1**	0.2312
**Alpelisib+Palbociclib-G2**	0.2095
**Alpelisib+Palbociclib-G3**	0.2604
**Alpelisib+Palbociclib-G4**	0.1906
**Duvelisib+Palbociclib-G1**	0.2415
**Duvelisib+Palbociclib-G2**	0.1460
**Duvelisib+Palbociclib-G3**	0.2377
**Duvelisib+Palbociclib-G4**	0.2658
**Quizartinib+Palbociclib-G1**	0.1976
**Quizartinib+Palbociclib-G2**	-
**Quizartinib+Palbociclib-G3**	0.1684
**Quizartinib+Palbociclib-G4**	-

Table S 4: Growth rate of MOLM-13 at day 2 under different treatments

**Treatment-generation**	**Growth rate**
**Control-G1**	0.1290
**Control-G2**	0.1674
**Control-G3**	0.1694
**Control-G4**	0.2125
**Gilteritinib-G1**	0.0186
**Gilteritinib-G2**	0.0210
**Gilteritinib-G3**	0.0577
**Gilteritinib-G4**	0.0176
**Abemaciclib-G1**	0.0222
**Abemaciclib-G2**	0.0254
**Abemaciclib-G3**	0.0230
**Abemaciclib-G4**	0.0174
**Gilteritinib+Palbociclib-G1**	0.0226
**Gilteritinib+Palbociclib-G2**	0.0216
**Gilteritinib+Palbociclib-G3**	0.0215
**Gilteritinib+Palbociclib-G4**	0.0212
**Gilteritinib+Abemaciclib-G1**	0.0224
**Gilteritinib+Abemaciclib-G2**	0.0269
**Gilteritinib+Abemaciclib-G3**	0.0251
**Gilteritinib+Abemaciclib-G4**	0.0177
**Abemaciclib+Copanlisib-G1**	0.0242
**Abemaciclib+Copanlisib-G2**	0.0264
**Abemaciclib+Copanlisib-G3**	0.0291
**Abemaciclib+Copanlisib-G4**	0.0184
**Abemaciclib+Quizartinib-G1**	0.0215
**Abemaciclib+Quizartinib-G2**	0.0209
**Abemaciclib+Quizartinib-G3**	0.0171
**Abemaciclib+Quizartinib-G4**	0.0244

Table S 5: Growth rate of MOLM-14 at day 2 under different treatments

**Treatment-generation**	**Growth rate**
**Control-G1**	0.3373
**Control-G2**	0.2852
**Control-G3**	0.4583
**Control-G4**	0.4265
**Gilteritinib-G1**	0.0895
**Gilteritinib-G2**	0.0209
**Gilteritinib-G3**	0.2474
**Gilteritinib-G4**	0.2125
**Abemaciclib-G1**	0.0267
**Abemaciclib-G2**	0.0169
**Abemaciclib-G3**	0.0211
**Abemaciclib-G4**	0.0485
**Gilteritinib+Palbociclib-G1**	0.0201
**Gilteritinib+Palbociclib-G2**	0.0245
**Gilteritinib+Palbociclib-G3**	0.0167
**Gilteritinib+Palbociclib-G4**	0.0212
**Gilteritinib+Abemaciclib-G1**	0.0224
**Gilteritinib+Abemaciclib-G2**	0.0269
**Gilteritinib+Abemaciclib-G3**	0.0251
**Gilteritinib+Abemaciclib-G4**	0.0177
**Abemaciclib+Copanlisib-G1**	0.0242
**Abemaciclib+Copanlisib-G2**	0.0264
**Abemaciclib+Copanlisib-G3**	0.0291
**Abemaciclib+Copanlisib-G4**	0.0184
**Abemaciclib+Quizartinib-G1**	0.0215
**Abemaciclib+Quizartinib-G2**	0.0209
**Abemaciclib+Quizartinib-G3**	0.0171
**Abemaciclib+Quizartinib-G4**	0.0244

Figure S8: The effect of combination treatments on CDK4 expression levels. MOLM-14 (a) and MV4-11 (b). Cells were cultured in the absence or presence of the indicated drugs for 24 h. Cell lysates were analysed by immunoblotting for the expression of CDK4. β-Actin was used as loading control. Adjusted densities based on (a) and (b) was calculated using the Fiji software and are shown in (c), in which x-axis positions from 1 to 11 represent the lanes in (a) and (b) from left to right.

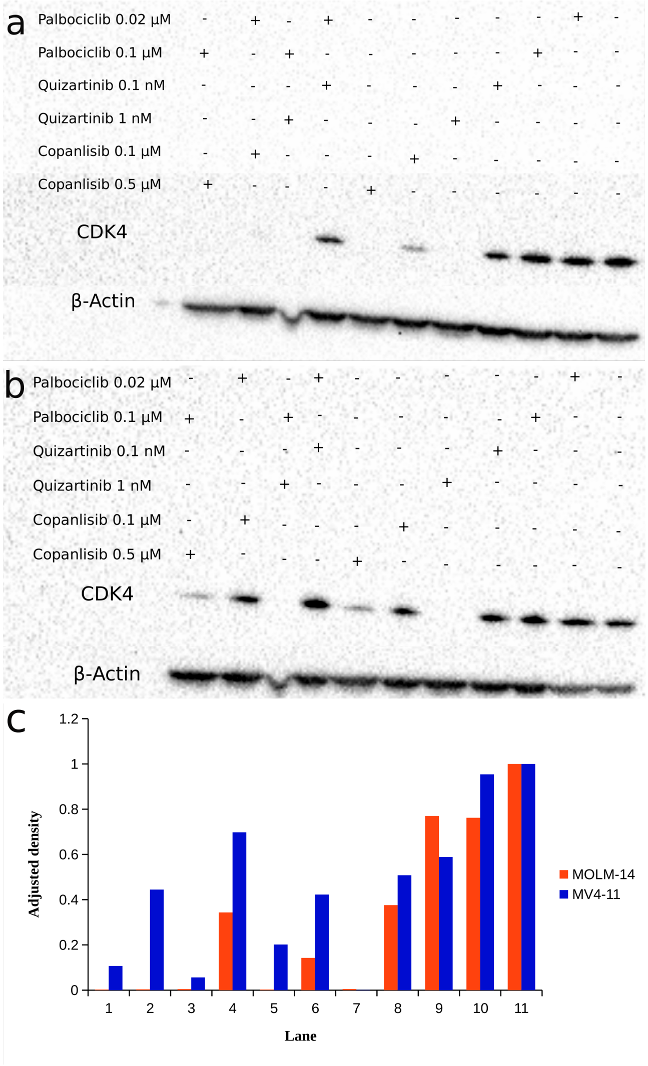

Figure S9: The effect of combination treatment on p-Akt expression levels. MOLM-14 (a) and MV4-11 (b). Cells were cultured in the absence or presence of the indicated drugs for 24 h. Cell lysates were analysed by immunoblotting for the expression of phosphorylated-Akt. β-Actin was used as loading control. Adjusted densities based on (a) and (b) were calculated using the Fiji software and showed on (c), in which, x-axis from 1 to 11 represented the lanes in (a) and (b) from left to right.

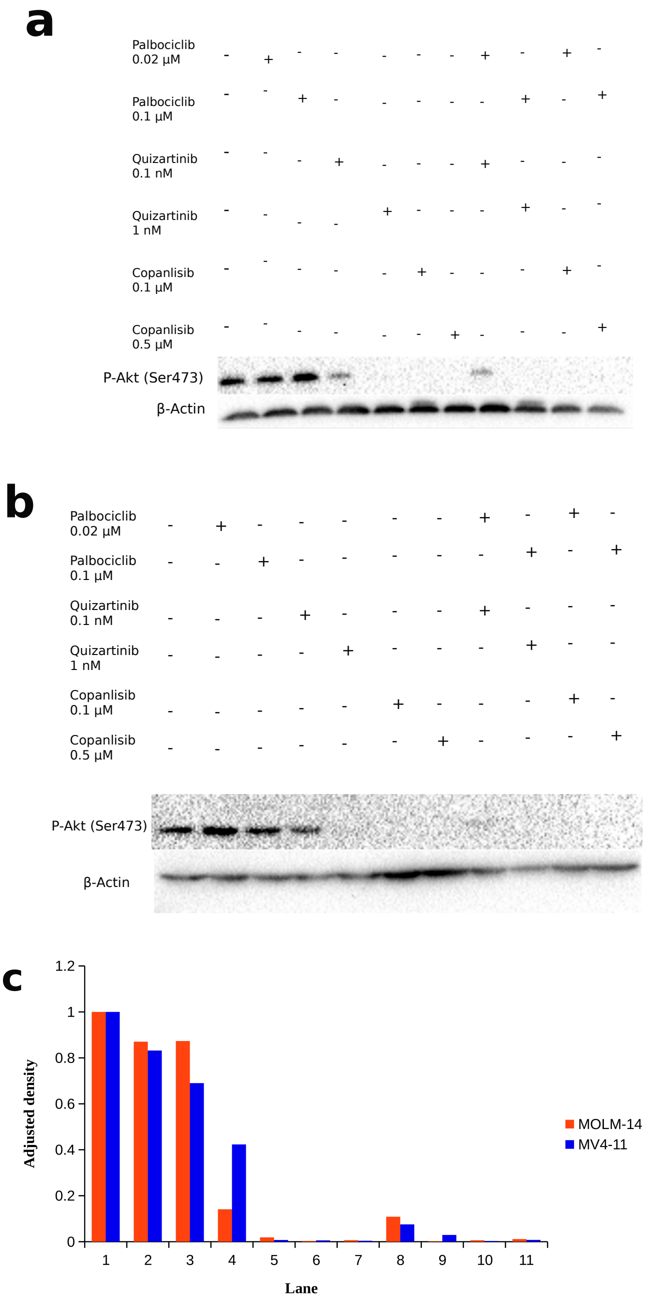

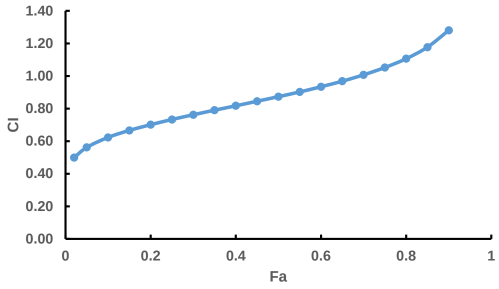

((a)) CI values measured in MOLM-13 cells after 48 hours in the combination of idelalisib and palbociclib

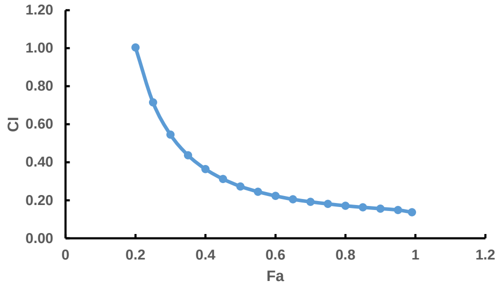

((b)) CI values measured in MOLM-14 cells after 48 hours in the combination of idelalisib and palbociclib

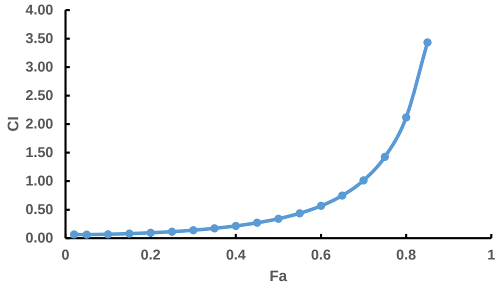

((c)) CI values measured in MV4-11 cells after 48 hours in the combination of idelalisib and palbociclib

Figure S10: CI values measured in AML cell lines after 48 hours in the combination of idelalisib and palbociclib at different concentrations.

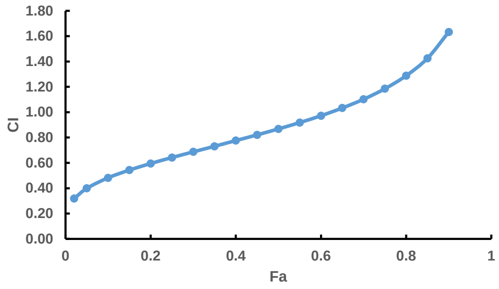

((a)) CI values measured in MOLM-13 cells after 48 hours in the combination of alpelisib and palbociclib

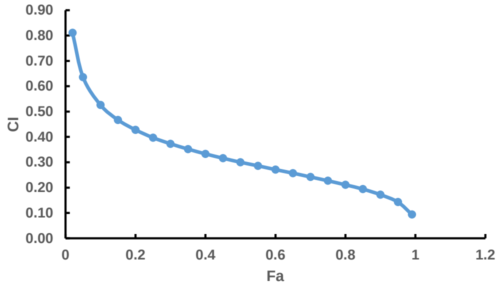

((b)) CI values measured in MOLM-14 cells after 48 hours in the combination of alpelisib and palbociclib

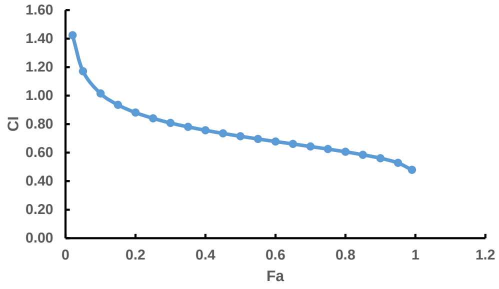

((c)) CI values measured in MV4-11 cells after 48 hours in the combination of alpelisib and palbociclib

Figure S11: CI values measured in AML cell lines after 48 hours in the combination of alpelisib and palbociclib at different concentrations.

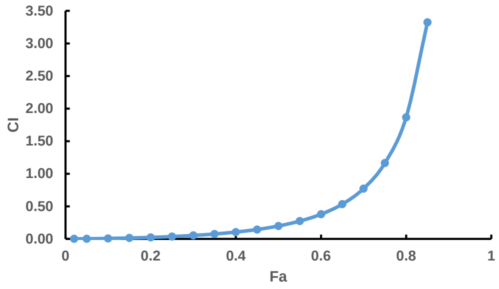

((a)) CI values measured in MOLM-13 cells after 48 hours in the combination of Copanlisib and palbociclib

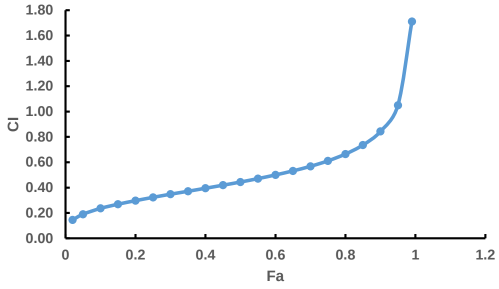

((b)) CI values measured in MOLM-14 cells after 48 hours in the combination of Copanlisib and palbociclib

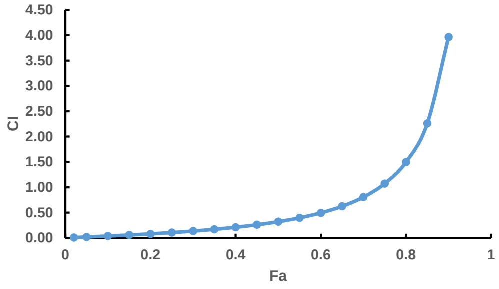

((c)) CI values measured in MV4-11 cells after 48 hours in the combination of Copanlisib and palbociclib

Figure S12: CI values measured in AML cell lines after 48 hours in the combination of Copanlisib and palbociclib at different concentrations.

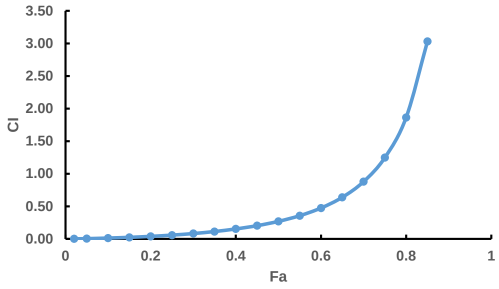

((a)) CI values measured in MOLM-13 cells after 48 hours in the combination of Duvelisib and palbociclib

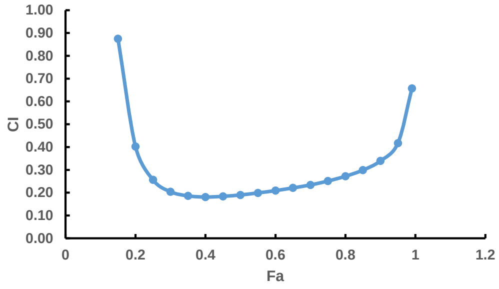

((b)) CI values measured in MOLM-14 cells after 48 hours in the combination of Duvelisib and palbociclib

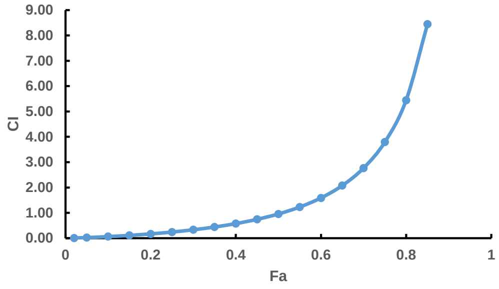

((c)) CI values measured in MV4-11 cells after 48 hours in the combination of Duvelisib and palbociclib

Figure S13: CI values measured in AML cell lines after 48 hours in the combination of Duvelisib and palbociclib at different concentrations.

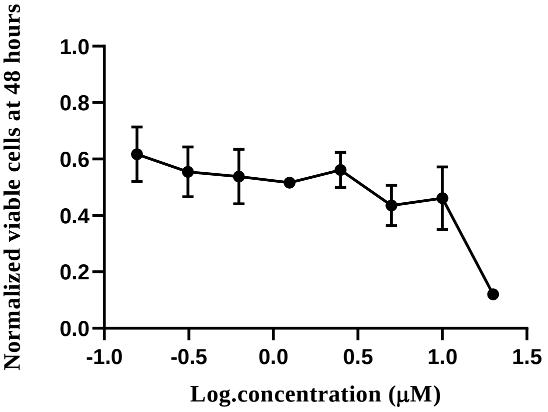

((a)) Normalized cell viability of MOLM-13 after 48 hours in various concentrations of abemaciclib

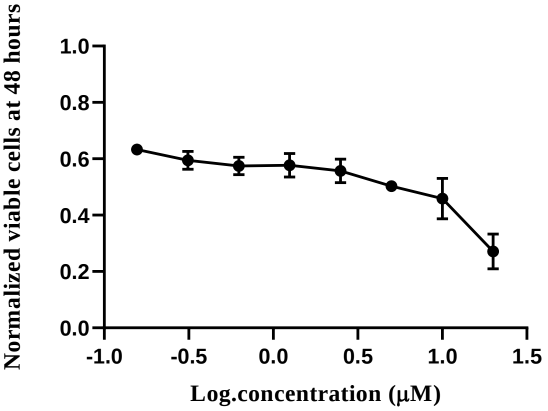

((b)) Normalized cell viability of MOLM-14 after 48 hours in various concentrations of abemaciclib

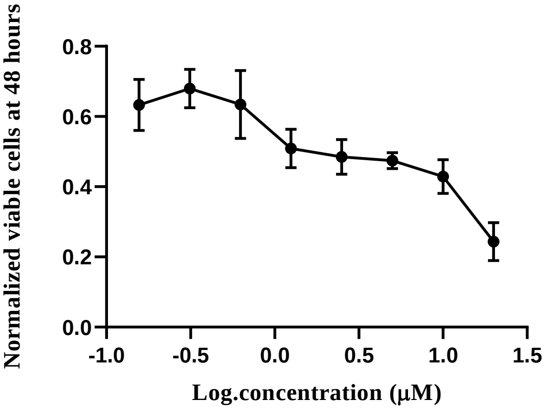

((c)) Normalized cell viability of MV4-11 after 48 hours in various concentrations of abemaciclib

Figure S14: Dose-response curve with CDK6 inhibitor abemaciclib alone in the AML cell lines. Three independent experiments were carried out.

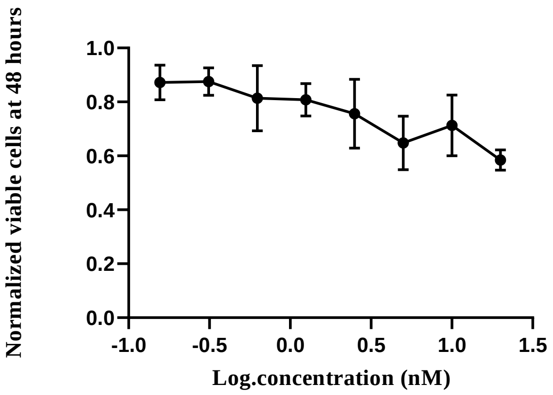

((a)) Normalized cell viability of MOLM-13 after 48 hours in various concentrations of gilteritinib

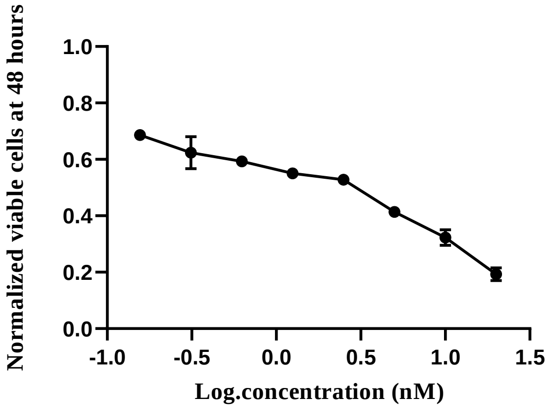

((b)) Normalized cell viability of MOLM-14 after 48 hours in various concentrations of gilteritinib

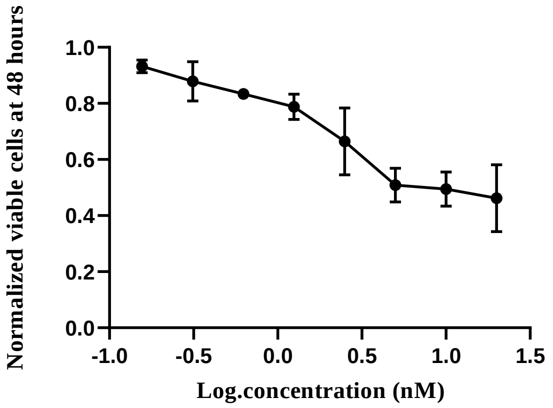

((c)) Normalized cell viability of MV4-11 after 48 hours in various concentrations of gilteritinib

Figure S15: Dose-response curve with FLT3 inhibitor gilteritinib alone in the AML cell lines. Three independent experiments were carried out.

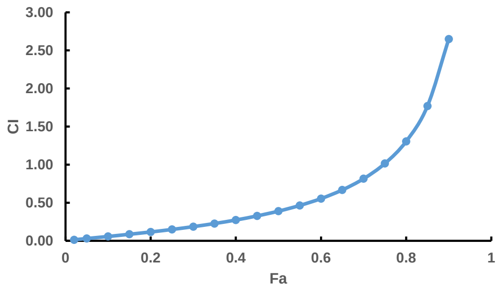

((a)) CI values measured in MOLM-13 cells after 48 hours in the combination of abemaciclib and copanlisib

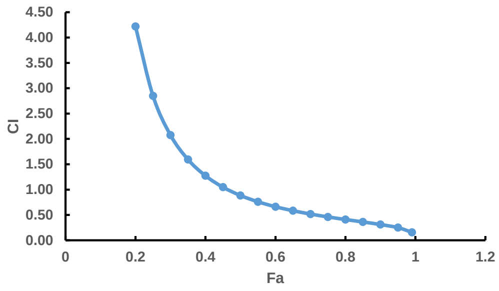

((b)) CI values measured in MOLM-14 cells after 48 hours in the combination of abemaciclib and copanlisib

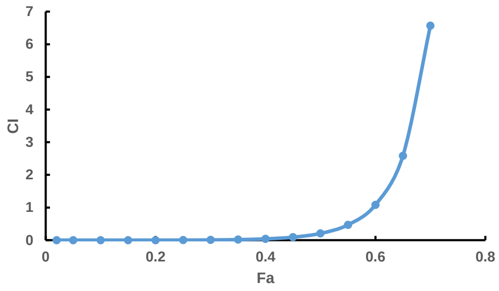

((c)) CI values measured in MV4-11 cells after 48 hours in the combination of abemaciclib and copanlisib

Figure S16: CI values measured in AML cell lines after 48 hours in the combination of abemaciclib and copanlisib at different concentrations.

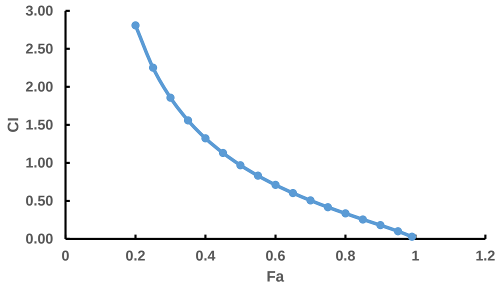

((a)) CI values measured in MOLM-13 cells after 48 hours in the combination of abemaciclib and gilteritinib

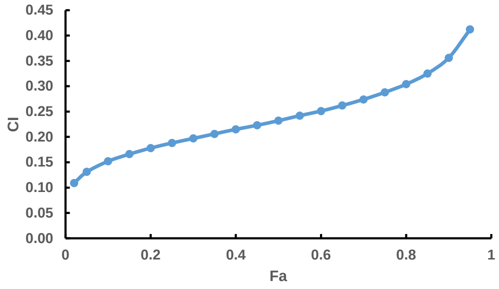

((b)) CI values measured in MOLM-14 cells after 48 hours in the combination of abemaciclib and gilteritinib

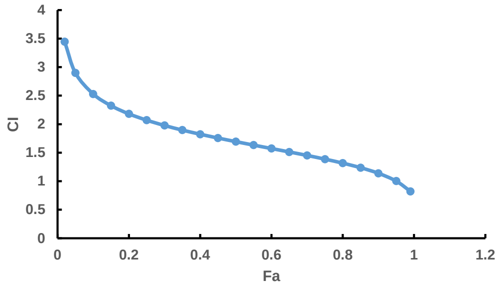

((c)) CI values measured in MV4-11 cells after 48 hours in the combination of abemaciclib and gilteritinib

Figure S17: CI values measured in AML cell lines after 48 hours in the combination of abemaciclib and gilteritinib at different concentrations.

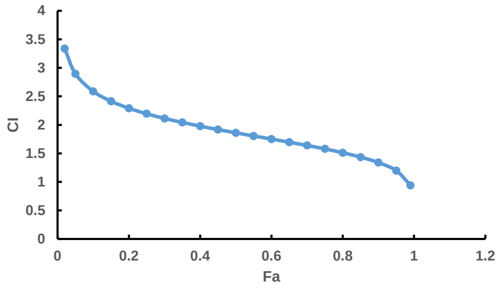

((a)) CI values measured in MOLM-13 cells after 48 hours in the combination of abemaciclib and quizartinib

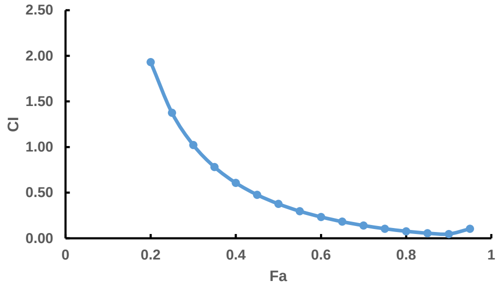

((b)) CI values measured in MOLM-14 cells after 48 hours in the combination of abemaciclib and quizartinib

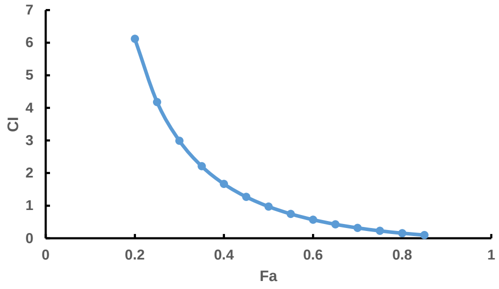

((c)) CI values measured in MV4-11 cells after 48 hours in the combination of abemaciclib and quizartinib

Figure S18: CI values measured in AML cell lines after 48 hours in the combination of abemaciclib and quizartinib at different concentrations.

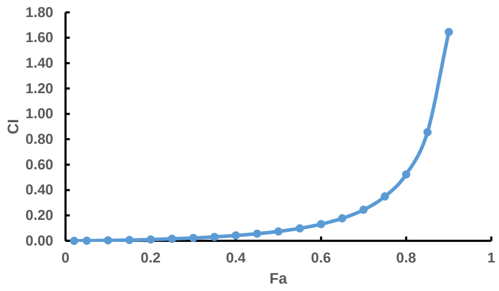

((a)) CI values measured in MOLM-13 cells after 48 hours in the combination of gilteritinib and copanlisib

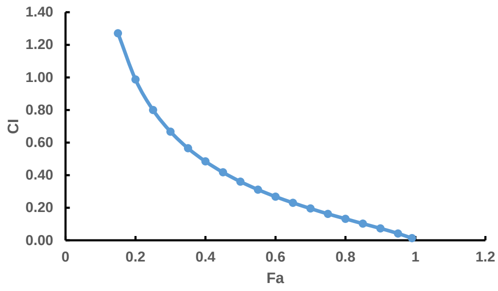

((b)) CI values measured in MOLM-14 cells after 48 hours in the combination of gilteritinib and copanlisib

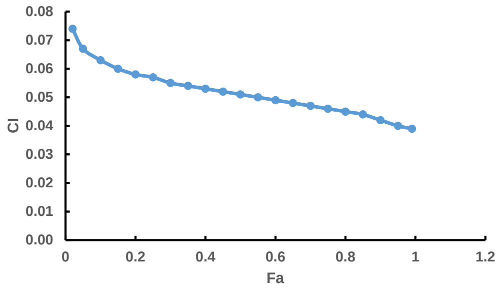

((c)) CI values measured in MV4-11 cells after 48 hours in the combination of gilteritinib and copanlisib

Figure S19: CI values measured in AML cell lines after 48 hours in the combination of gilteritinib and copanlisib at different concentrations.

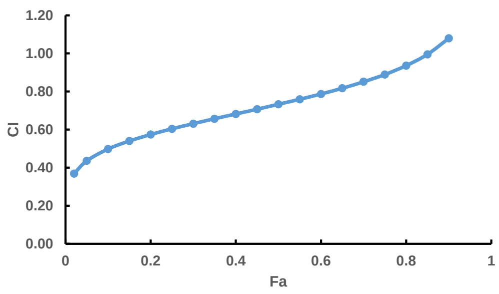

((a)) CI values measured in MOLM-13 cells after 48 hours in the combination of gilteritinib and palbociclib

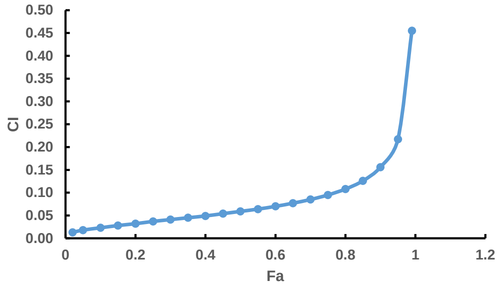

((b)) CI values measured in MOLM-14 cells after 48 hours in the combination of gilteritinib and palbociclib

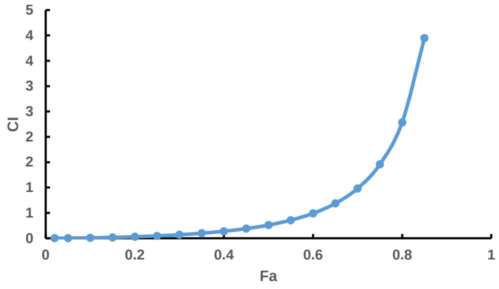

((c)) CI values measured in MV4-11 cells after 48 hours in the combination of gilteritinib and palbociclib

Figure S20: CI values measured in AML cell lines after 48 hours in the combination of gilteritinib and palbociclib at different concentrations.

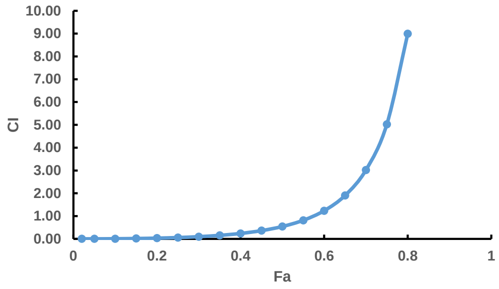

((a)) CI values measured in MOLM-13 cells after 48 hours in the combination of quizartinib and palbociclib

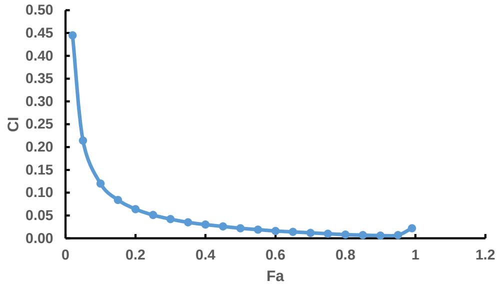

((b)) CI values measured in MOLM-14 cells after 48 hours in the combination of quizartinib and palbociclib

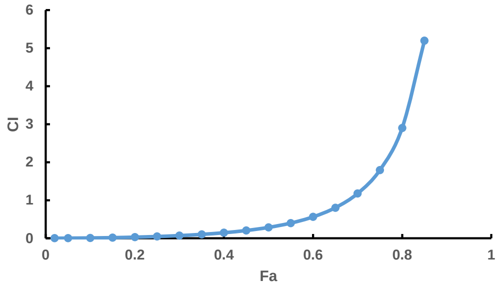

((c)) CI values measured in MV4-11 cells after 48 hours in the combination of quizartinib and palbociclib

Figure S21: CI values measured in AML cell lines after 48 hours in the combination of quizartinib and palbociclib at different concentrations.

Figure S22: Normalised cell viabilities of MOLM-13 (a, d, g), MOLM-14 (b, e, h) and MV4-11 (c, f, i) cells after 48 hours of treatment with alpelisib (grey lines, a, b and c), duvelisib (grey lines, d, e, and f) or idelalisib (grey lines, g, h, and i). Dark blue lines in all subfigures represented the combination of palbociclib with the PI3K inhibitor and red lines represent cells treated with palbociclib. In all subfigures, the x-axis on the bottom shows the concentration of the PI3K inhibitor while the x-axis on the top shows the concentration of palbociclib. Error bars represent standard deviations calculated from three measurements.

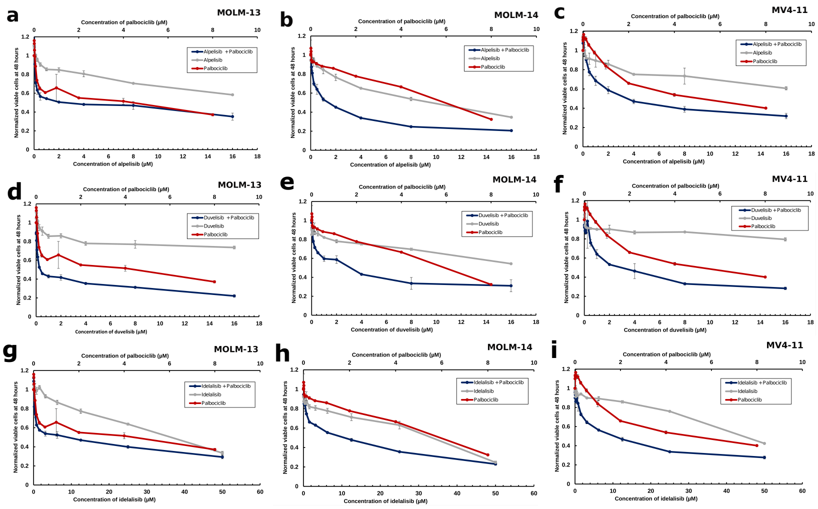

Figure S23: Cell growth of AML cell lines- MOLM-13 (a, d, g, j), MOLM-14 (b, e, h, k) and MV4-11 (c, f, i, l)- treated by palbociclib, idelalisib (a, b, c) or a combination thereof; palbociclib, copanlisib (d, e, f) or a combination thereof; palbociclib, duvelisib (g, h, i) or a combination thereof; palbociclib, alpelisib (j, k, l) or a combination thereof. Error bars were calculated from four experiments. ANOVA with Tukey's post hoc test: one asterisk indicates $p < 0.05$ between experimental group and control, two asterisks indicate $p < 0.01$, three asterisks indicate $p < 0.001$ while ns indicates $p > 0.05$. The concentrations of the inhibitors match their IC30, Table 4.

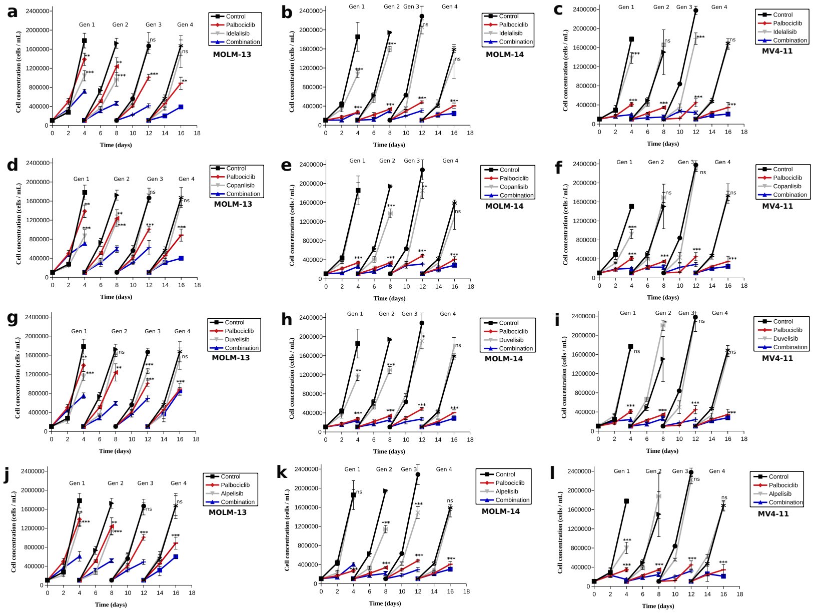

Figure S24: Normalised cell viabilities of MOLM-13 (a, b, c), MOLM-14 (d, e, f) and MV4-11 (g, h, i) cells after 48 hours treated with palbociclib (red lines, a, d and g), copanlisib (red lines, d, e, and h) or abemaciclib (red lines, c, f, and i). While dark blue lines in all subfigures represent the combination of gilteritinib with each different inhibitor and grey lines represent cells treated with gilteritinib. In all subfigures, the x-axis on the bottom shows the concentration of gilteritinib while the x-axis on the top shows the concentration of indicated inhibitor. Error bars represent standard deviations calculated from three measurements.

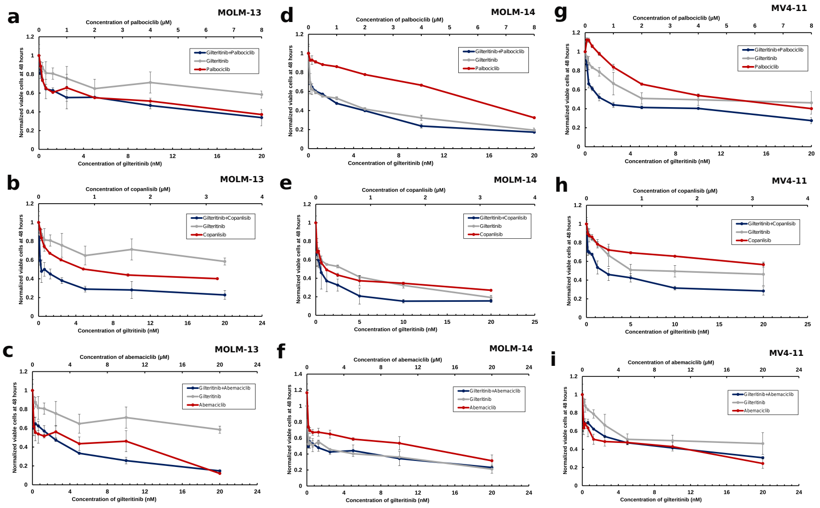

Figure S25: Normalised cell viabilities of MOLM-13 (a, b), MOLM-14 (c, d) and MV4-11 (e,f) cells after 48 hours of treatment with copanlisib (red lines, a, c and e), quizartinib (red lines, b, d and f) or their combinations with abemaciclib. Dark blue lines in all subfigures represented the combination of abemaciclib with different inhibitors and grey lines represented cells treated with abemaciclib. In all subfigures, the x-axis on the bottom shows the concentration of abemaciclib while the x-axis on the top shows the concentration of indicated inhibitor. Error bars represent standard deviations calculated from three measurements.

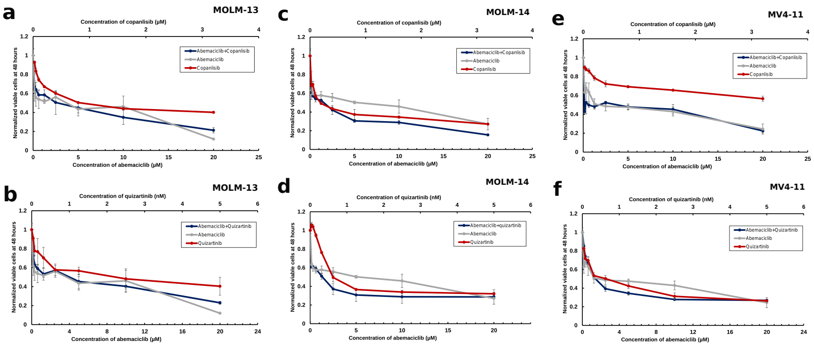

Supplement: Supplementary file 1 — Additional file 1. Additional Figures S1–S25, Tables S1–S5. [file 12935_2023_3000_MOESM1_ESM.pdf]
